# Commanding or Being a Simple Intermediary: How Does It Affect Moral Behavior and Related Brain Mechanisms?

**DOI:** 10.1523/ENEURO.0508-21.2022

**Published:** 2022-10-14

**Authors:** Emilie A. Caspar, Kalliopi Ioumpa, Irene Arnaldo, Lorenzo Di Angelis, Valeria Gazzola, Christian Keysers

**Affiliations:** 1 Social Brain Lab, Netherlands Institute for Neuroscience, Royal Netherlands Academy of Arts and Sciences, 1105 BA, Amsterdam, The Netherlands; 2 The Moral & Social Brain Lab, Department of Experimental Psychology, Ghent University, B-9000 Ghent, Belgium; 3 Department of Psychology, University of Amsterdam, 1018 WT, Amsterdam, The Netherlands

**Keywords:** empathy for pain, hierarchy, moral behavior, obedience, responsibility, sense of agency

## Abstract

Psychology and neuroscience research have shown that fractioning operations among several individuals along a hierarchical chain allows diffusing responsibility between components of the chain, which has the potential to disinhibit antisocial actions. Here, we present two studies, one using fMRI (Study 1) and one using EEG (Study 2), designed to help understand how commanding or being in an intermediary position impacts the sense of agency and empathy for pain. In the age of military drones, we also explored whether commanding a human or robot agent influences these measures. This was done within a single behavioral paradigm in which participants could freely decide whether or not to send painful shocks to another participant in exchange for money. In Study 1, fMRI reveals that activation in social cognition-related and empathy-related brain regions was equally low when witnessing a victim receive a painful shock while participants were either commander or simple intermediary transmitting an order, compared with being the agent directly delivering the shock. In Study 2, results indicated that the sense of agency did not differ between commanders and intermediary, no matter whether the executing agent was a robot or a human. However, we observed that the neural response over P3 event-related potential was higher when the executing agent was a robot compared with a human. Source reconstruction of the EEG signal revealed that this effect was mediated by areas including the insula and ACC. Results are discussed regarding the interplay between the sense of agency and empathy for pain for decision-making.

## Significance Statement

In hierarchical situations, one person decides and orders, and another person executes. In the present study, we use MRI and EEG to investigate the neurocognitive processes altered in hierarchical chains to explain how being in the position of commander or in the position of intermediary impacts moral behaviors. Results showed that in the two positions, empathy for the pain of others is altered compared with being the agent directly delivering the shock. The sense of agency does not differ between commanders and intermediaries. These results show how powerful hierarchical situations can facilitate the commission of actions that harm others, as agency and empathy are split across multiple individuals.

## Introduction

Numerous historical examples have shown the power of fractioning operations across different individuals to facilitate atrocious acts of mass annihilation (De Swaan, 2015). A common example of fractioning operations are hierarchical situations: a superior communicates a plan and a subordinate executes it. The superior then bears responsibility for the decision but is distanced from the outcomes, while the subordinate experiences authorship over the action but may experience reduced responsibility for its outcomes (Bandura, 2006). In many organizations, orders are embedded in an even longer chain of commands in which a given commander often merely relays the orders received from a superior. Commanders can thus also be intermediaries, an aspect that diffuses the psychological responsibility for the decisions to inflict harm. Experimental research has shown that such intermediary positions increase obedience to orders to hurt someone compared with being the author of that action or being the person giving the orders (Kilham and Mann, 1974; Milgram, 1974). However, the neural mechanisms by which being in the intermediary position or being in the commanding position disinhibit harming others remain largely unknown and represent the main focus of the present article. In addition, modern warfare increasingly replaces the human soldiers that were at the bottom of the hierarchical chain and ultimately caused the harm to the enemy with artificial agents (e.g., drones, missiles, robots; Di Nucci and Santoni de Sio, 2016). How this affects the experience of commanders remains poorly understood.

Despite the lack of experimental research on the neurocognitive processes associated with moral behavior for intermediaries and commanders, previous scientific literature on the position of subordinate (i.e., the agent) has brought some evidence that at least two processes could be involved in how hierarchy influences the willingness to harm: sense of agency (SoA) and empathy for pain.

The SoA refers to the feeling that we are the authors of, and thus potentially responsible for, our actions and their consequences in the external world (Gallagher, 2000). It is often measured implicitly through the intentional binding effect (Moore and Haggard, 2010): participants have to estimate the duration of the time interval between an action (e.g., pressing a button) and its consequences (e.g., hearing the beep it produces), with cases in which participants experience a stronger sense of agency leading to shorter time estimates (Moore and Haggard, 2010). The relationship between time perception and sense of agency is thought to be mediated by striatal dopaminergic activity, which is crucial for time perception (Meck, 2006) and is also driving information from basal ganglia to frontal motor areas (Nachev et al., 2008), key brain regions in generating the sense of agency. The feeling of responsibility is a related, but more explicit and social concept (Balconi, 2010), commonly evaluated with explicit questions asked of the participants (Li et al., 2011). Previous studies have shown that being in a position of the subordinate (or “agent”) executing an action commanded by an experimenter or induced by a computer reduces the sense of agency and the feeling of being responsible for an action (Barlas and Obhi, 2013; Caspar et al., 2016, 2018, 2020b). Other studies have also shown that asking participants to remember situations in which they had a low social power reduced their sense of agency compared with when they had a high social power (Obhi et al., 2012). Such results thus suggest that the sense of agency is reduced when people have reduced power in social situations.

Empathy for pain is a fundamental process that allows us to understand and imagine what others feel by processing their pain within our own pain system. An extensive literature has indeed shown that seeing another individual in pain triggers an empathic response in the brain of the observer (Keysers and Gazzola, 2014; Decety, 2011; Lamm et al., 2019; Singer and Bernhardt, 2012). The intensity of empathic experience has typically been measured through subjective reports, but neuroscientists have increasingly supplemented these reports with measurements of the degree of activation in regions involved in pain experience while participants witness the pain of others. In particular, functional magnetic resonance imaging (fMRI) studies have revealed that two nodes of the putative “pain matrix” (Iannetti and Mouraux, 2010; i.e., the brain regions recruited while participants experience pain on their own body) are also recruited while witnessing the pain of others: the anterior cingulate cortex (ACC) and the anterior insula (AI; for a recent meta-analysis, see Jauniaux et al., 2019; Lamm et al., 2011; Timmers et al., 2018). The exact contributions of the AI and ACC to witnessing the pain of others remains unclear, but rodent studies have shown the existence of neuron homologs of the ACC that encode both the intensity of the pain of others and pain in the self (Carrillo et al., 2019), and deactivating the ACC leads to a reduction in distress in animals witnessing the distress of others (Han et al., 2020). In particular, when rodents had to choose between two actions leading to rewards, with one also leading to pain in another animal, deactivating the ACC reduced the sensitivity to the pain caused to others (Hernandez-Lallement et al., 2020). With regard to the anterior insula, animal studies demonstrating the causal contribution of the region to the pain of others remain rare, but there is evidence that witnessing the pain of others increases brain activity in the insula (Sakaguchi et al., 2018), and deactivating the insula reduces the hyperalgesia observed when rodents cohabit with animals in pain (Zaniboni et al., 2018). Intracortical electroencephalography (EEG) recordings have also revealed that neural activity in the insula correlates with subjective reports of pain intensity while participants witness other individuals’ facial expressions of pain or their hands receiving noxious stimulation (Soyman et al., 2021). Apart from literature relating specific regions with vicarious pain, in recent years there have been studies (Krishnan et al., 2016; Zhou et al., 2020) identifying whole-brain vicarious pain-predictive patterns. Using multivariate pattern analysis to fMRI data acquired during the observation of painful stimulations in others, weighted brain maps that respond to pain observation with a sensitive and specific way have been contracted. In EEG studies, early potentials (Early Automatic Component, N200; reflecting a bottom-up emotional sharing response) and late potentials (LPPs; representing a subsequent top-down evaluative response; Chen et al., 2014) are sensitive to the witnessing of a painful stimulus being delivered to another individual. Similar to the sense of agency, being in a position of subordinate executing an action commanded by an experimenter reduces the empathic response to the pain of others (Caspar et al., 2020b; Lepron et al., 2015). Another study has shown that being reminded about a situation in which individuals had high social power increased the empathic neural response to painful pictures compared with being reminded about a low social power condition (Galang et al., 2021). These studies suggest that being in a position with low decisional power also impacts empathy for pain. Finally, Cui et al. (2015) showed that if the participant’s action is not the only cause for a victim’s pain, neural signatures of empathy are reduced when witnessing the pain of the victim compared with cases in which the participant’s actions are the only cause for that pain.

However, these studies never directly compared the positions of commanding or being a simple intermediary in a single paradigm, thus preventing direct comparisons between those two social positions (Lepron et al., 2015; Caspar et al., 2016, 2018, 2020b). Further, in some of those studies, a participant’s actions or decisions were not measured, and participants were simple observers (Galang et al., 2021; Obhi et al., 2012). The present study aimed to fill this gap by comparing how commanding or being in an intermediary position impacts the sense of agency and empathy for pain within a single behavioral paradigm in which participants could decide or had to follow the orders to send or not send painful shocks to another participant in exchange for money.

Participants were recruited in pairs and respectively played the role of the person giving orders or the role of the “victim.” When they were in the role of the person giving orders, participants had to give an order to an agent to send or not to send a real, mildly painful electric shock to the victim in exchange for a small monetary gain, which increased their own remuneration for their participation in the study. In that position, participants were either free to decide which order to send to the agent (i.e., they were “commanders”) or were given an order by the experimenter that they had to transmit to the agent (i.e., they were “intermediaries”). When participants were in the commander position, we also modulated the entity executing their orders: they were either giving orders to another human or to a nonhumanoid robot. Past studies showed that SoA is reduced when participants believe that they are, or actually are, performing a task with another human (Beyer et al., 2017, 2018; Caspar et al., 2018). It has been argued that SoA is reduced in the presence of another intentional agent because it elicits a representation of their potential actions, a phenomenon called “vicarious agency” (Berberian et al., 2012), while this is not the case for nonagentic or nonintentional agents (Ciardo et al., 2020). Past literature showed that robots can be perceived as agentic entities (Wang and Quadflieg, 2015). However, this perception is reduced when the robot is a computer or a nonhumanoid robot (Sahaï et al., 2022). In the present study, as nonhumanoid robots are considered entities with low or no intentionality at all, we wanted to understand whether giving orders to a robot in the intermediary position would boost the sense of agency compared with giving orders to another human being, on which they can diffuse their own responsibility (Bandura et al., 1999).

In a first study (Study 1), we used fMRI to investigate how the processing of the pain felt by the victim for each shock received is modulated by the different experimental conditions by quantifying BOLD signals in regions associated with empathy, while the participant witnessed the shock being delivered versus not being delivered. In a second study (Study 2), we used electroencephalography to further explore how the different experimental conditions modulated the pain processing, as measured by the amplitude of the N200, P3, and late positive potentials (Coll, 2018), and the sense of agency, as measured by intentional binding effects on time interval estimation. The sense of agency was not measured using time interval estimation in Study 1 because, to separate brain activity related to motor response from those related to processing the pain of the victim in fMRI, long action–outcome intervals (i.e., between 2.5 and 6 s) have to be used (Caspar et al., 2020b; Cui et al., 2015), which are too long for the measurement of the sense of agency with the method of interval estimates. Previous studies indeed showed that if the consequence of an action occurs >4 s after the action, modulations of the sense of agency no longer lead to measurable changes in time perception (Humphreys and Buehner, 2009). We therefore used electroencephalography, which has a better temporal resolution than fMRI, in Study 2 to investigate the two targeted neurocognitive processes in a single paradigm.

Based on past literature (Galang et al., 2021; Obhi et al., 2012), we expected that being in a low-social power position (i.e., intermediaries) would reduce empathy for pain and the sense of agency compared with a position of higher power (i.e., commanders). This may be even more the case when orders are transmitted to another human compared with a condition in which orders are transmitted to a robot, as responsibility can be diffused between two humans, but less in the case of a human–robot interaction (Ciardo et al., 2020). In a former study, results indicated that in hierarchical situations, the sense of agency was reduced for both the commander and the agent executing orders (Caspar et al., 2018). In the present study, we did not use an experimental condition in which people would also be in the position of the agent, as it would have considerably increased the testing time. However, we compared the results from Study 1 with those from a recent fMRI study using a matching design to empathy for pain for participants playing the role of the subordinate (i.e., referred to as agents in the study by Caspar et al., 2020b). This comparison allowed us to understand how empathy for the pain of the victim is modulated through three different hierarchical positions: commander, intermediary, and agent. We also further conducted exploratory analyses to investigate how self-reported personality traits influenced prosocial behaviors.

## Materials and Methods: Study 1 (MRI)

### Participants

Forty participants were recruited in 20 dyads, based on the number of participants recruited in the study by Caspar et al. (2020b). None of the participants reported to know each other. The full dataset of two participants was excluded from all the analyses because of disobeying by contradiction (performing the reverse action from what they were ordered). fMRI data from one participant were removed from the fMRI analyses because of extensive movements, 1 because of scanner failure; 12 because they delivered fewer than five shocks in each condition, causing too few a number of repetitions to measure reliable signals; and 1 because of orders systematically disobeyed by administering shocks even when requested not to do so, which resulted in no No-Shock trials. These resulted in 23 participants for the fMRI (6 males; mean ± SD age, 24.26 ± 3.17 years) and 38 for the behavioral analyses (13 males; mean ± SD age, 25.05 ± 3.6 years). The study was approved by the local ethics committee of the University of Amsterdam (Project 2017-EXT-8298). Data are made available on OSF (https://osf.io/scw9z/).

### Procedure and material

Upon arrival in the laboratory, both participants received instructions about the experiment and provided informed consent together, ensuring that they were each aware of the other’s consent. Then, their individual pain threshold for the electrical stimulation was determined, as described in the study by Caspar et al. (2016). Two electrodes were placed on the participants’ left hand on the abductor pollicis muscle to produce a clear and visible muscle twitch, and the threshold was increased by steps of 1 mA until a mildly painful stimulation was achieved. The pain threshold was determined by asking a series of questions to the participants about their pain perception during the calibration (1. “Is it uncomfortable?; 2. “Is it painful?”; 3. “Could we increase the threshold?”).

Participants were assigned to start either as “participant” or victim by randomly picking up a card in a box, but were offered the possibility to change if they wanted to. The participant who was in the role of the commander was placed in the MRI scanner to perform the task, while the participant assigned to the role of the victim was seated at a table in the nearby control room.

Victims were asked to place their left hand on a black sheet positioned in the field of view of the camera and not to move their hand during the entire scanning session. The victim was invited to watch a neutral documentary to make the time pass.

**Figure 1.**
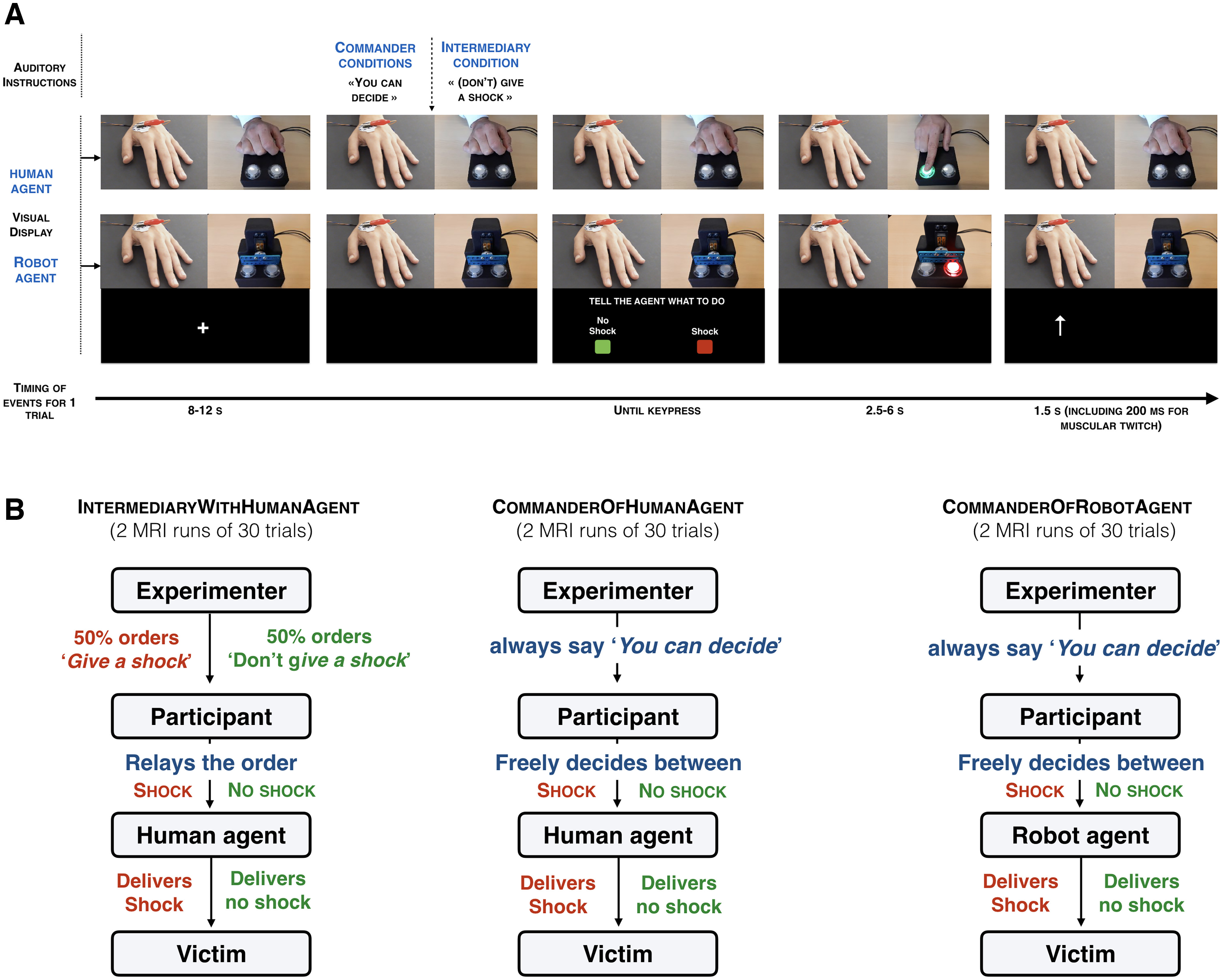
***A***, Visual display of the structure of a single trial. Participants inside the MRI scanner had two real-time video feedbacks, one from the victim’s left hand with the electrodes connected to the shock machine and one with the agent (human or robot) and its button box. When the participants pressed either the SHOCK or the NO SHOCK button, the corresponding buttons on the agent’s button box appeared in red or green. The agent then pressed on the colored button. An arrow pointing to the top then appeared on the screen to remind participants to look at the victim’s hand at that moment. If the SHOCK button was pressed, participants could see a visible muscular twitch on the victim’s hand. ***B***, Schematic representation of each experimental condition. Each participant underwent 2 runs of 30 trials in the scanner. In the two IntermediaryWithHumanAgent runs, in half the trials they heard the experimenter tell them to “give a shock” and then pressed a red button relaying this order to the human agent that they could then see press the corresponding red button and the victim’s hand then twitched. In the other half of the trials, the experimenter told them “don’t give a shock,” and then had to relay that order by pressing the green NO SHOCK button, leading the agent to press the green button as well. They could then see the victim not receiving a shock. In the four commander runs, they heard the experimenter tell them “you can decide,” and the participant in the scanner was then in a position of commander, freely deciding whether to shock or not shock on each trial. In two of these runs, the agent was again a human (CommaderOfHumanAgent). In the other two runs, the human agent was replaced by a mechanical device that pressed the ordered button (CommanderOfRobotAgent).

To be able to compare the MRI data acquired in this experiment with the MRI data from a previous study (Caspar et al., 2020b) in which participants played the role of the agents (i.e., agents), we preserved the exact same trial structure. Each trial started with a jittered fixation cross lasting 8–12 s (Fig. 1). Then, participants heard a verbal instruction from the experimenter in all three experimental conditions (i.e., CommanderOfHumanAgent, CommanderOfRobotAgent, and IntermediaryWithHumanAgent). In the IntermediaryWithHumanAgent condition, the experimenter told participants to either “give a shock” or “don’t give a shock.” To have a similar causation and auditory information in all the experimental conditions, participants also received a verbal instruction in the two commander conditions. This verbal instruction was “you can decide.” Participants were told that the experimenter would give those instructions from outside the scanner room through the interphone during the two commander conditions and from inside the scanner room during the IntermediaryWithHumanAgent condition. In reality, these sentences were prerecorded to keep control of the precise timing of each event during the scanning session. To increase the authenticity of the procedure, each sentence was recorded six times with small variations in the voice to generate credible variance, and these recordings were presented in random order. In addition, the audio recordings included a background sound similar to interphone communications. Participants were also told that during the IntermediaryWithHumanAgent condition, the experimenter would wear a microphone to increase the intensity of her voice to overcome the noise of the MRI scanner. The experimenter explicitly exhibited herself at the beginning of the IntermediaryWithHumanAgent condition by speaking with the participant laying down inside the scanner, but then moved to the corner of the room to avoid visual interference because of her presence.

After receiving the verbal order, a picture of two rectangles, a red one labeled “SHOCK” and a green one labeled “NO SHOCK,” was displayed on the left and right bottom of the screen. The key-outcome mapping varied randomly across trials to concentrate motor preparatory activity in the interval between choice screen onset and key-press, but the outcome was always fully congruent with the participant’s decision (i.e., the agent never disobeyed the order given by the participant). Participants could then press one of the two buttons to ask the agent to execute their order. On the right top of the screen, participants could see the agent pressing a button corresponding to the order they had given. The agent had a button box with two transparent buttons. After commanders gave their order, the corresponding button popped in the corresponding color (red, SHOCK; green, NO SHOCK) so that the agent knew which button to press. This procedure ensured that participants could track the agent’s action. It also ensured that the agent was pressing the correct button corresponding to the requested order. Pressing the SHOCK button delivered a shock to the victim, while pressing the NO SHOCK button did not deliver any shocks. The shock was delivered between 2.5 and 6 s after the key-press (Cui et al., 2015; Caspar et al., 2020b). For the participants to also track the consequence of their orders, another real-time camera feed displayed on the left top of the screen showed the victim’s hand, with electric shocks eliciting a visible muscle twitch. Seven hundred fifty milliseconds before the display of the shock, an arrow pointing to the top was displayed to remind participants to look at the video. This arrow also appeared when the NO SHOCK button had been pressed to keep a similar structure in all trials. That arrow disappeared 750 ms after. To further encourage participants to pay attention to the victim’s hand, on six trials in each MRI run a pain rating scale appeared, ranging from “not painful at all” (0) to “very painful” (1000). Participants were asked to rate the intensity of the shock (or no sock) seen on the last trial by moving the red marker bar along the scale using four buttons. The keys below the middle fingers allowed to modify the number associated with the position of the marker by steps of ±100. The keys below the index fingers allowed modification of the answer by steps of ±1. After a fixed duration of 6 s, their answer was saved and the next trial started. If no shocks were delivered on that trial, participants were asked to report that the shock was “not painful at all.”

In the IntermediaryWithHumanAgent, participants were asked to obey the experimenter’s orders and to transmit those orders to the agent. In the commander conditions, participants were entirely free to decide which order to send to the agent. In the CommanderOfHumanAgent condition, the agent was a human, a confederate of the experimenter. Participants were told that the human agent was part of the experimenter’s team. In the CommanderOfRobotAgent condition, the agent was a robot. The experimenter confirmed that agents would always obey the commander’s order in all the experimental conditions.

The task was split into six MRI runs of 30 trials each, two runs for the CommanderOfHumanAgent condition, two runs for the CommanderOfRobotAgent condition, and two runs for the IntermediaryWithHumanAgent condition (presented in six separate fMRI acquisition runs). The order of the experimental conditions was counterbalanced across participants. Anatomical images were recorded between the fourth and fifth runs of fMRI acquisition. At the end of each task run, participants rated their explicit sense of responsibility over the outcomes of their actions on an analog scale presented on the screen, ranging from “not responsible at all” to “fully responsible.” Each delivered shock was rewarded with +€0.05 in all the experimental conditions, and in the IntermediaryWithHumanAgent run, participants were instructed to transmit an order to shock on 50% of trials.

At the end of the experimental session, participants were asked to fill out eight questionnaires assessing several personality traits. Those questionnaires included the following: (1) the Interpersonal Reactivity Index (Davis et al., 1980); (2) the Short Dark Triad (Jones and Paulhus, 2014); (3) the Levenson Self-Report Psychopathy Scale (Levenson et al., 1995); (4) the Moral Foundation Questionnaire (Graham et al., 2011); (5) the Aggression-Submission-Conventionalism scale (ASC; Dunwoody and Funke, 2016); (6) the Right-Wing Authoritarianism scale (Altemeyer, 1983); (7) Hypomania Checklist (Angst et al., 2005); and (8) a debriefing assessing what they felt during the experiment. Participants were paid separately, based on their own gain during the experiment.

### General data analyses

Data were analyzed with both frequentist and Bayesian statistics (Dienes, 2011), except for voxelwise brain analyses that were only analyzed using frequentist approaches. Bayesian statistics assess the likelihood of the data under both the null and the alternative hypotheses. In most cases, we report the Bayesian equivalent (BF_10_), which corresponds to the *p*(data|*H*_1_)/*p*(data|*H*_0_). Generally, a BF value between one-third and three indicates that the data are similarly likely under the *H*_1_ and *H*_0_, and that the data thus do not adjudicate which is more likely. A BF_10_ value below one-third or above three is interpreted as supporting *H*_0_ and *H*_1_, respectively. For instance, BF_10_ = 20 would mean that the data are 20 times more likely under *H*_1_ than *H*_0_, providing very strong support for *H*_1_, while BF_10_ = 0.05 would mean that the data are 20 times more likely under *H*_0_ than *H*_1_, providing very strong support for *H*_0_ (Marsman and Wagenmakers, 2017). BF and *p* values were calculated using JASP (Love et al., 2019, p. 2019) and the default priors were implemented in JASP (Keysers et al., 2020).The default priors used in JASP depend on the statistical tests performed (for ANOVA, see Rouder et al., 2012; for *t* tests, see Ly et al., 2016; for correlations, see Wagenmakers et al., 2016). In cases where a one-tailed hypothesis was tested, the directionality of the hypothesized effect is indicated as a subscript to the BF (e.g., BF_+0_ for a positive effect, BF_–0_ for a negative effect).

### fMRI

MRI images were recorded using a 3 tesla scanner (Ingenia CX System, Philips) and a 32-channel head coil. T1-weighted structural images were recorded with the following specifications: matrix = 240 × 222; 170 slices; voxel size = 1 × 1 × 1 mm. Six runs of functional images were recorded [matrix (M) × pixel (P): 80 × 78; 32 transversal slices in ascending order; TR = 1.7 s; TE = 27.6 ms; flip angle = 72.90°; voxel size = 3 × 3 × 3 mm; slice gap = 0.349 mm). Images were acquired in ascending order.

### General fMRI data processing and first-level contrasts

MRI data processing was conducted in SPM12 (Ashburner et al., 2014). EPI images were slice time corrected to the middle slice and realigned to the mean EPI image. High-quality T1-weighted images were coregistered to the mean EPI image and segmented. The normalization parameters computed during the segmentation were used to normalize the gray matter segment (1 × 1 × 1 mm) and the EPI images (2 × 2 × 2 mm) to the MNI templates. Afterward, images were smoothed with a 6 mm kernel.

At the first level, we defined separate regressors for Shock (S) and No-Shock (NS) trials, with the three different conditions modeled in separate runs to identify the activations associated with witnessing pain. Each of these regressors started 750 ms before the moment of the shock, which lasted 250 ms, up to 500 ms after the moment of the shock. This moment corresponded to when the arrow pointing to the video feedback appeared, to remind participants to watch the screen displaying the victim’s hand. The same 1.500 ms time window was taken for Shock and No-Shock trials. Additional regressors included the following: (1) the auditory orders from the experimenter (starting between 8 and 12 s after the start of the trial) together with the button presses (participants could press the key whenever they wanted right after the auditory orders) and the presses of the (human or robot) agent; and (2) the pain rating scale (appearing on 6 of 30 trials randomly 1 s after the arrow pointing toward the video feedback disappeared) together with the responsibility rating scale (appearing at the end of each MRI run, 1 s after the arrow pointing toward the video feedback disappeared or again 1 s after the pain scale). Trials where participants disobeyed were modeled in additional regressors of no interest separately for “prosocial” disobedience (i.e., they refused to administer a shock while having been ordered to send a shock) and “antisocial” disobedience (i.e., they administered a shock while having been ordered not to send a shock). Finally, six additional regressors of no interest were included to model head translations and rotations.

At the first level, we defined the following three main contrasts of interest: [CommanderOfHumanAgent(S-NS)–IntermediaryWithHumanAgent(S-NS)], [CommanderOfRobotAgent(S-NS)–IntermediaryWithHumanAgent(S-NS)], and [CommanderOfRobotAgent(S-NS)–CommanderOfHumanAgent(S-NS)], on which we then computed a random-effect one-sample *t* test at the group level of analyses.

### Vicarious pain signatures analyses

Associating changes in brain activity in a single location with specific mental processes entails issues of reverse inference (Poldrack, 2006). For this reason, in addition to our standard GLM approach, we used the multivariate physical vicarious pain signature developed by Zhou et al. (2020) that scales selectively with perceiving observed pain while witnessing body parts in pain. This signature map was developed by training a multivariate pattern classifier on images of body parts in pain and was used to quantify empathic responses while seeing the sight of a hand receiving shocks in our study. Since our contrasts of interest were CommanderOfHumanAgent, CommanderOfRobotAgent, and IntermediaryWithHumanAgent, we brought the signature into our fMRI analysis space using ImageCalc and then dot multiplied the parameter estimate volumes for each participant and for each of these contrasts with the weight map of the signature. The result was a value per participant per contrast that indicated the loading of the contrast on the signature. The result was a value per participant per contrast that indicated the loading of the contrast on the signature (i.e., the dot multiplication of the fMRI parameter estimate volume with the signature). We then brought the data from this loading into JASP, compared them against zero, and compared the conditions of the current study with each other and with the AgentFree and AgentCoerced conditions from the study by Caspar et al. (2020b).

## Results: Study 1

### Number of shocks delivered

**Figure 2.**
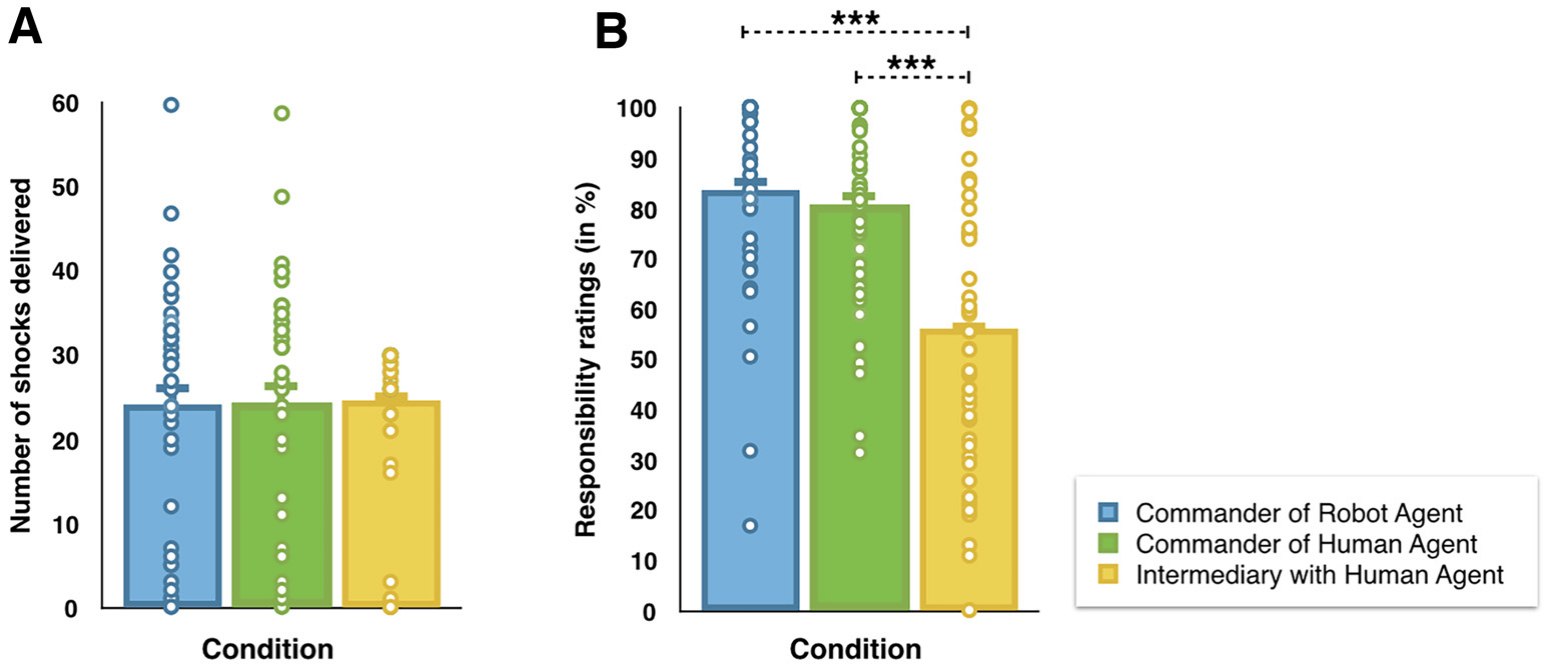
***A***, Graphical representation of the number of shocks delivered in the three experimental conditions. ***B***, Graphical representation of responsibility ratings in the three experimental conditions. All tests were two tailed. ***p ≤ 0.001; BF_10_ > 3. Errors bars represent standard errors.

In the commander conditions, participants could freely decide which order to send to the agent. In the CommanderOfHumanAgent condition, participants asked the human agent to administer 24.34 of 60 shocks (SD = 15.43; minimum, 0 shocks; maximum, 59 shocks) to the victim. In the CommanderOfRobotAgent condition, participants asked the robot agent to administer 24.13 of 60 shocks (SD = 15.13; minimum, 0 shocks; maximum, 60 shocks) to the victim. In the IntermediaryWithHumanAgent condition, the experimenter ordered delivery of shocks on 30 of the 60 trials. Of these, the participants relayed the shock order to the human agent on average 24.63 of 30 trials (SD = 9.003; minimum, 0 trials; maximum, 30 trials), while in the remaining trials, they disobeyed and ordered the agent not to deliver a shock. More specifically, of the 40 participants, 12 reported that they voluntarily disobeyed the orders of the experimenter on some trials. Among those 12 participants, 10 disobeyed “prosocially” by refusing to send a shock during “shock trials” and by telling the agent not to deliver a shock to the victim even if the experimenter asked them to do so (i.e., prosocial disobedience), and 2 disobeyed “by contradiction”; that is, they disobeyed as often on “don’t shock” trials and on “shock trials.” We conducted a repeated-measures ANOVA with Condition (CommanderOfHumanAgent, CommanderOfRobotAgent, and IntermediaryWithHumanAgent) as the within-subject factor and Role Order (commander first, victim first) as the between-subject factor on the number of shocks sent in each experimental condition. Results indicated that none of the main effects or their interactions were significant (all *p *>* *0.3; Fig. 2*A*). The Bayesian version of the same analysis indicated that the main effect of Condition and the interaction Condition × Order of the Role were strongly in favor of *H*_0_ (BF_incl_ = 0.062 and BF_incl_ = 0.033, respectively). The main effect of the Order of the Role was slightly in favor of *H*_0_ (BF_incl_ = 0.387).

Regarding the distribution of the data points, we also performed Levene’s tests to test the equality of variance between each experimental condition. Results indicated that variability was reduced in the IntermediaryWithHumanAgent condition compared with the CommanderOfRobotAgent condition (*F*_(1,70)_ = 13.897, *p *<* *0.001) and compared with the CommanderOfHumanAgent condition (*F*_(1,70)_ = 17.507, *p *<* *0.001). The CommanderOfRobotAgent and the CommanderOfHumanAgent conditions did not differ (*p *>* *0.7).

### Responsibility ratings

At the end of each experimental condition, participants had to report how responsible they felt for the outcome of their orders. We conducted a repeated-measures ANOVA with Condition (CommanderOfHumanAgent, CommanderOfRobotAgent, and IntermediaryWithHumanAgent) as the within-subject factor and Role Order (Commander first, Victim first) as the between-subject factor on the responsibility ratings. Both the frequentist and the Bayesian results supported a main effect of Condition (*F*_(2,74)_ = 25.038, *p *<* *0.001, η^2^_partial_ = 0.404, BF_incl_ = 4.597E + 6; Fig. 2*B*). Paired comparisons indicated that responsibility ratings were higher in the CommanderOfRobotAgent condition (82%; 95% CI = 74.5–89.5) than in the IntermediaryWithHumanAgent condition (56.2%; 95% CI = 46.8–65.6; *t*_(38)_ = −5.455; *p *<* *0.001; Cohen’s *d* = –0.873; BF_10_ = 5822.75) and in the CommanderOfHumanAgent condition (81%; 95% CI = 74.4–87.4) than in the IntermediaryWithHumanAgent condition (56.2%; 95% CI = 46.8-65.6); *t*_(39)_ = −5.050, *p *<* *0.001, Cohen’s *d* = –0.799, BF_10_ = 1889.49). The difference in responsibility ratings between the CommanderOfHumanAgent and the CommanderOfRobotAgent conditions was inconclusive (*p *>* *0.1, BF_10_ = 0.466). The main effect of Role Order (*p *=* *0.063; BF_incl_ = 0.968) and the interaction Condition × Role Order (*p *>* *0.5, BF_incl_ = 0.439) were inconclusive.

### Pain scale

We did not analyze the pain ratings as only nine participants had a full dataset in all the experimental conditions. As the pain scale appeared randomly, for several participants it never appeared after a Shock or a No-Shock trial in at least one experimental condition, thus precluding the conducting of repeated-measures ANOVA.

### fMRI whole-brain analyses

**Figure 3.**
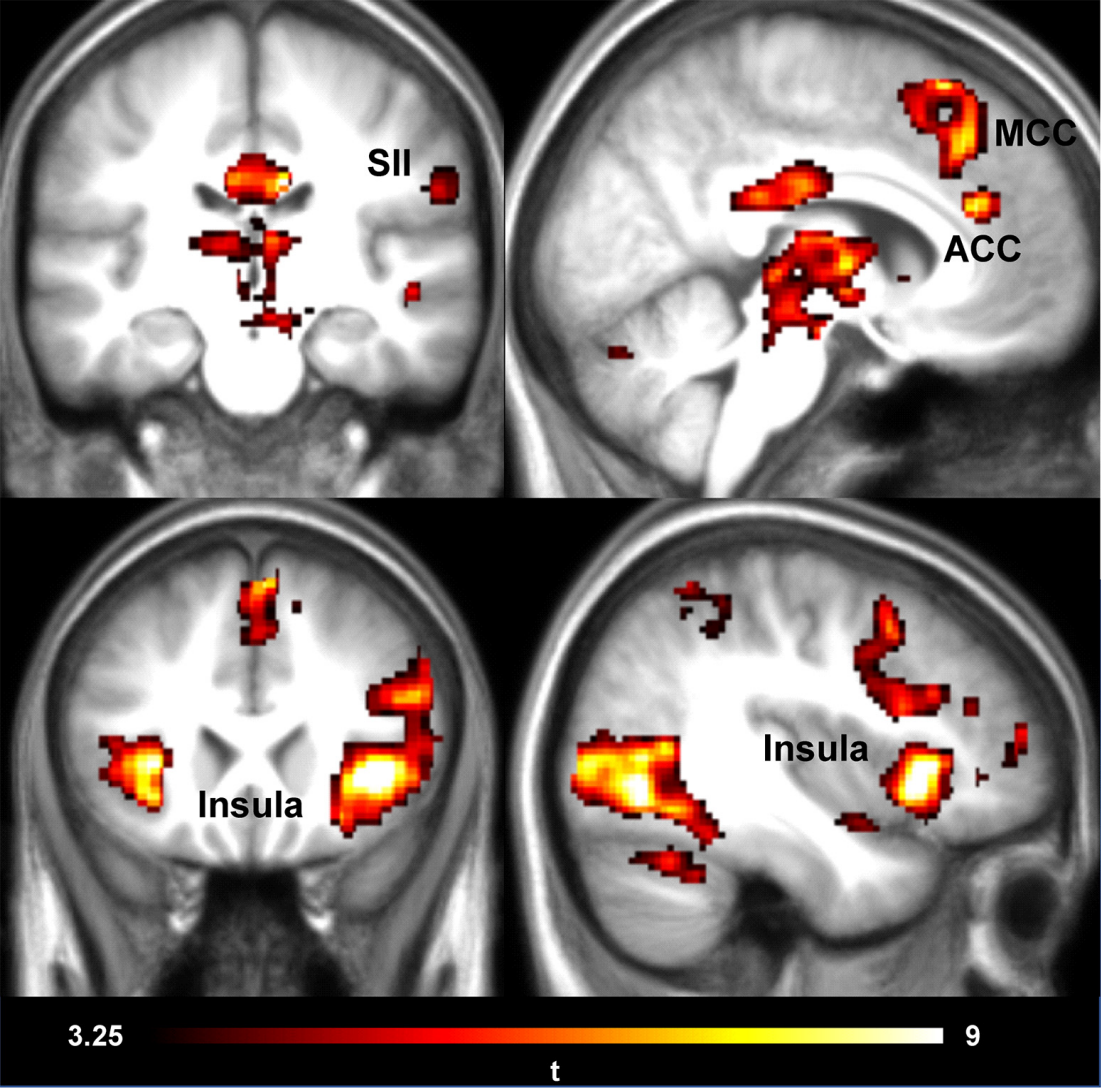
MRI results. Shock–No-Shock contrast for all experimental conditions together. Peak coordinates can be seen in Extended Data Figure 3-1. Results are shown thresholded using *p*_FWE_ < 0.05 at cluster level (cFWE = 160 voxels) following a cluster-cutting threshold at *t* = 3.5 and *p *<* *0.001.

We first ensured that we could detect the vicarious pain activation network in our study, including especially the AI and the ACC. We thus computed a main Shock–No-Shock contrast, regardless of the experimental condition. We observed the typical pain observation network activation, including the ACC, medial cingulate cortex, secondary somatosensory cortex (SII), and insula (Fig. 3, Extended Data Fig. 3-1), suggesting that witnessing the shock delivered to the victim’s hand indeed triggered an empathic neural response.

10.1523/ENEURO.0508-21.2022.f3-1 Figure 3-1 BOLD activity of Shock–No-Shock contrast for all experimental conditions together. Only clusters surviving a 5% FWE correction at the cluster size are reported (*t* = 3.5; *p* < 0.001; cluster size 160). Brain regions are identified using the Anatomy Toolbox (Eickhoff et al., 2005). Download Figure 3-1, DOCX file.

Results showed that at the whole-brain level, none of our contrasts of interest [CommanderOfHumanAgent(S-NS)–IntermediaryWithHumanAgent(S-NS)], [CommanderOfRobotAgent(S-NS)–IntermediaryWithHumanAgent(S-NS)], and [CommanderOfRobotAgent(S-NS)–CommanderOfHumanAgent(S-NS)] showed significant results in a random effects one-sample *t* test.

### Comparison between being commander and agent

One of the aims of this study was to compare the empathic neural response when participants are in the role of a commander (MRI data acquired in the present experiment) and when they are in the role of an agent executing an order of a commander (MRI data from a previous study by Caspar et al., 2020b). At the second level, we thus conducted five two-sample *t* tests comparing the two conditions of the previous agent study with the three conditions of the current commander study as follows: [AgentFree(S-NS)–CommanderOfRobotAgent(S-NS)], [AgentFree(S-NS)–CommanderOfHumanAgent(S-NS)], [AgentFree(S-NS)–IntermediaryWithHumanAgent(S-NS)], [AgentCoerced(S-NS)–CommanderOfRobotAgent(S-NS)], [AgentCoerced(S-NS)–CommanderOfHumanAgent(S-NS)], and [AgentCoerced(S-NS)–IntermediaryWithHumanAgent(S-NS)].

**Figure 4.**
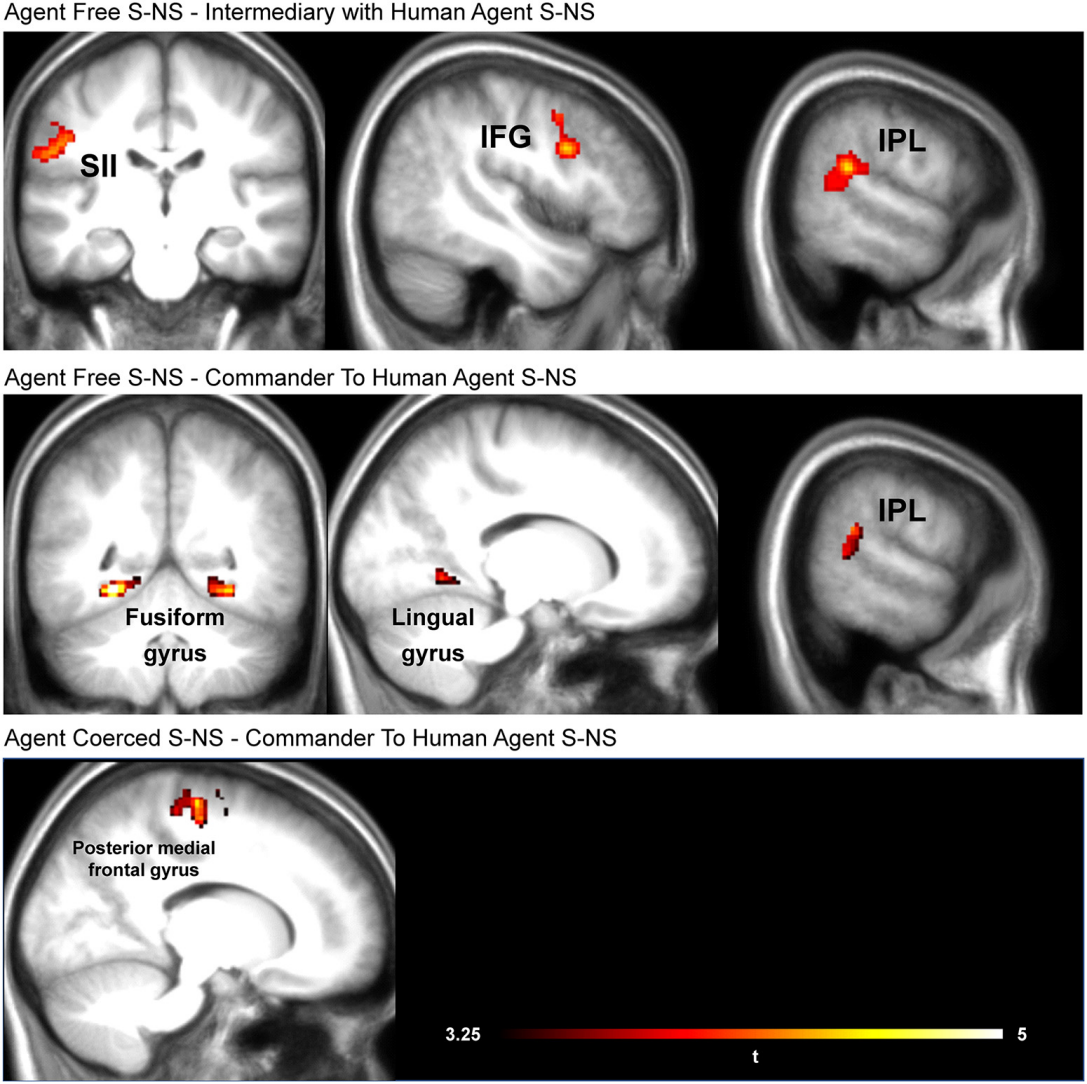
Results of two sample *t* tests between the conditions [AgentFree(S-NS)–IntermediaryWithHumanAgent(S-NS)]_FWE_ at cluster level (236 voxels; *t* = 3.5, *p *< 0.001), [AgentFree(S-NS)–CommanderOfHumanAgent(S-NS)]_FWE_ at cluster level (163 voxels; *t* = 3.5, *p *<* *0.001), and [AgentCoerced(S-NS)–CommanderOfHumanAgent(S-NS)]_FWE_ at cluster level (315 voxels; *t* = 3.5, *p *<* *0.001). Peak coordinates can be seen in Extended Data Figure 4-2. None of the reverse contrasts demonstrated any significant results. Extended Data Figure 4-2 displays the uncorrected maps.

Results on Figure 4 and Extended Data Figure 4-1 were thresholded at *p*_unc_ < 0.001 and 5% familywise error (FWE) corrected at the cluster level and significant activation was observed for the [AgentFree(S-NS)–IntermediaryWithHumanAgent(S-NS)], [AgentFree(S-NS)–CommanderOfHumanAgent(S-NS)], and [AgentCoerced(S-NS)–CommanderOfHumanAgent(S-NS)].

10.1523/ENEURO.0508-21.2022.f4-1 Figure 4-1 BOLD activity of comparisons between the agent and commander study conditions. Only clusters surviving a 5% FWE correction at the cluster size are reported (*t* = 3.5; *p* < 0.001; cluster size, 160). Brain regions are identified using the Anatomy Toolbox (Eickhoff et al. 2005). Download Figure 4-1, DOCX file.

10.1523/ENEURO.0508-21.2022.f4-2 Figure 4-2 Results of two-sample *t* tests between the conditions CommanderOfRobotAgent(S-NS)–IntermediaryWithHumanAgent(S-NS) and CommanderOfRobotAgent(S-NS)–CommanderOfHumanAgent(S-NS) uncorrected (3.5 < *t* < 5; *p *<* 0*.005). Download Figure 4-2, TIF file.

### Vicarious pain signatures

**Figure 5.**
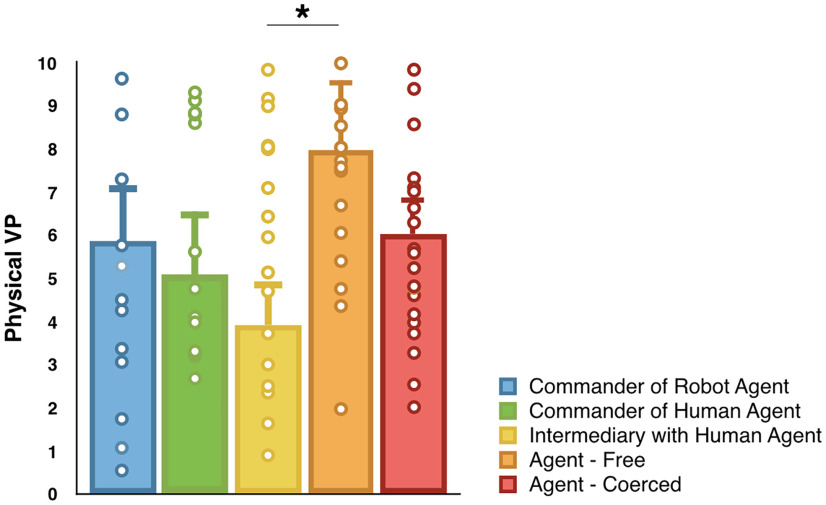
Results from the neurologic physical vicarious pain signature analysis in arbitrary units for the conditions Agent Free S-NS, Agent Coerced S-NS, IntermediaryWithHumanAgent S-NS, CommanderOfHumanAgent S-NS, and CommanderOfRobotAgent S-NS. Each signature was significantly different from 0 (all *p* values < 0.002, all BF_10_ values > 17). All comparisons were two tailed. Errors bars represent standard errors.

To examine whether the lack of difference between our three conditions (i.e., CommanderOfHumanAgent, CommanderOfRobotAgent, and IntermediaryWithHumanAgent) was because of the strict criteria of mass multivariate testing in fMRI and, to explore more specifically whether the manipulation influences empathic brain responses, we leverage the multivariate physical vicarious pain signature developed by Zhou et al. (2020) to quantify empathic responses while seeing body parts in pain. We chose this particular signature, because it was trained on images of body parts in pain that best approximates the sight of hand receiving shocks in our study. Figure 5 shows the differential response (Shock–No-Shock) of this signature in the conditions of the current study and in the study by Caspar et al. (2020b). As expected, in all cases, the differential response was significantly positive. At the second level, we thus conducted six two-sample *t* tests comparing the two conditions of the previous agent study with the three conditions of the current commander study as follows: AgentFree(S-NS)–CommanderOfRobotAgent(S-NS) (*t*_(52)_ = 1.195, *p *= 0.237, Cohen’s *d* = 0.327, BF_10_ = 0.496), AgentFree(S-NS)–CommanderOfHumanAgent(S-NS) (*t*_(52)_ = 1.546, *p *=* *0.128, Cohen’s *d* = 0.423, BF_10_ = 0.733), AgentFree(S-NS)–IntermediaryWithHumanAgent(S-NS) (*t*_(52)_ = 2.508, *p *=* *0.015, Cohen’s *d* = 0.686, BF_10_ = 3.444), AgentCoerced(S-NS)–CommanderOfRobotAgent(S-NS) (*t*_(52)_ = 0.108, *p *=* *0.914, Cohen’s *d* = 0.30, BF_10_ = 0.276), AgentCoerced(S-NS)–CommanderOfHumanAgent(S-NS) (*t*_(52)_ = 0.587, *p *=* *0.560, Cohen’s *d* = 0.160, BF_10_ = 0.317), and AgentCoerced(S-NS)–IntermediaryWithHumanAgent(S-NS) (*t*_(52)_ = 1.609, *p *=* *0.114, Cohen’s *d* = 0.440, BF_10_ = 0.795). These analyses revealed enhanced activation of the physical vicarious pain signature when agents were freely deciding compared with intermediates that were following orders and then delivering the same orders to a human agent. The other comparisons showed evidence for absence or close to evidence of absence of an effect. We additionally performed three paired-sample *t* tests comparing the three conditions of the current commander study: IntermediaryWithHumanAgent(S-NS)–CommanderOfHumanAgent(S-NS) (*t*_(21)_ = –0.723, *p *= 0.477, Cohen’s *d* = –0.151, BF_10_ = 0.277), IntermediaryWithHumanAgent(S-NS)–CommanderOfRobotAgent(S-NS) (*t*_(21)_ = −1.274, *p *=* *0.216, Cohen’s *d* =  –0.266, BF_10_ = 0.448), and CommanderOfHumanAgent(S-NS)–CommanderOfRobotAgent(S-NS) (*t*_(21)_ = –0.447, *p *=* *0.659, Cohen’s *d* = –0.093, BF_10_ = 0.240), which showed evidence for the absence of a difference among the conditions.

As an extra sanity check, we also computed the loading of the signature map on the Shock–No-Shock contrast regardless of the experimental condition and contrasted it against zero. As expected there was a significant positive effect (*t*_(22)_ = 5.921, *p *<* *0.001, Cohen’s *d* = 1.235, BF_10_ = 3546.471).

## Discussion: Study 1

In Study 1, we aimed to understand whether commanding or being in the position of the intermediary transmitting orders would influence how participants process the pain of a victim receiving mildly painful electric shocks. We also sought to understand how giving orders to another human being or to a robot would influence the same process. The BOLD signal clearly distinguished Shock and No-Shock trials in regions typically associated with empathy for pain, including cingulate, insular, and somatosensory brain regions (Jauniaux et al., 2019; Lamm et al., 2011). In a previous study, the authors assessed whether the observation of a virtual avatar in pain in a study like that by Milgram (1974) would trigger brain activity consistent with personal distress or empathic concern in the person sending the painful shocks (Cheetham et al., 2009). However, the authors did not observe the classical brain activation associated with affect sharing in the ACC and in the insula, while we observed such activations in the present study and in past studies (Caspar et al., 2020b). A critical difference is that in our study, our participants were delivering real pain to another real human participant instead of a fake virtual avatar.

Results from our fMRI analyses, however, did not reveal differences among our three conditions sufficiently strong to survive our *p* < 0.001 threshold. With *N* = 23 participants, and *p* < 0.001 voxelwise threshold, our study would require such differences to have a large effect size of *d* = 0.9 to be detected in 80% of cases so that our lack of significant difference suggests the absence of a large effect size of our manipulation. Smaller effect sizes of our manipulation cannot be excluded.

To explore the notion that all of the conditions tested here lead to reduced pain processing compared with directly being the agent, we ran additional comparisons between the remote conditions in the current experiment with previous data using a matching experimental design in which participants were the agent delivering the shocks (Caspar et al., 2020b). Specifically, when comparing the conditions in which participants were intermediaries (i.e., IntermediaryWithHumanAgent condition) to the condition in which participants were agents and free to decide, we observed higher activation when witnessing Shock versus No-Shock outcomes for the Free condition in SII, inferior frontal gyrus (IFG), and inner plexiform layer (IPL). The IFG, especially the dorsal part as in our results, and IPL form key elements of the network activated both while performing and observing hand actions (Gazzola and Keysers, 2009; Caspers et al., 2010), and both regions contain mirror neurons in monkeys (Kohler et al., 2002; Rozzi et al., 2008) and may have processed the hand movement signaling the delivery of the shock in our experiment. SII is part of the network responsive both while participants receive tactile stimulation on their own body and while observing other individuals receive similar tactile stimulation, and may have processed the tactile experiences of the other individual (Keysers et al., 2004, 2010). In a meta-analysis on pain empathy considering almost a 100 fMRI experiments by Jauniaux et al. (2019), the IFG and IPL were both found to be recruited while witnessing the pain of others. Additional evidence for different processing between these two conditions also came from our physical vicarious pain signature analysis, which revealed a significantly different loading on the physical vicarious pain signature between these conditions with the FreeAgent condition having a larger loading than the IntermediaryWithHumanAgent condition. We then compared the conditions in which participants could freely decide which orders to give to another human (i.e., CommanderOfHumanAgent) to the equivalent free situation, in which participants were agents and free to decide which button to press (i.e., Free condition; Caspar et al., 2020b). Results indicated that activity in areas IPL and fusiform gyrus, which have been linked in the literature with empathy and emotional social perception (Geday et al., 2003; Zaki et al., 2009; Janowski et al., 2013), was higher when participants were agents and could freely decide than when they were commanders and could freely decide. Comparing free agents and free commanders was interesting as the decisional power is the same for both roles when they are free to decide about the action to perform, but only agents execute the motor actions leading to the outcome. The differences in activation observed between free agents and free commanders thus suggest that performing the action engages more areas that are important for social cognition compared with having decisional power but being further away from the outcome of that same action.

Comparing the CommanderOfHumanAgent condition and the Agent Coerced (Caspar et al., 2020b) showed more activation for agents than for commanders in posterior medial frontal gyrus, an area that has been linked with cognitive control, response conflict, decision uncertainty, and cognitive dissonance (Ridderinkhof et al., 2004; Izuma et al., 2015). This suggests that in hierarchical situations, the agent is more engaged and experiencing more conflict for his actions, even coerced ones, than commanders giving orders. However, it is important to be aware of the limitations of reverse inference and the difficulty of unambiguously associating activity in specific brain regions with mental processes (Poldrack, 2006). Thus, these conclusions should be interpreted with caution.

All our participants acted both as agents and victims, in randomized order. To examine whether having been a victim altered the behavior of commanders compared with being commander first, we examined whether the number of shocks and the sense of responsibility was influenced by the order. In either case, we did not find evidence for an effect of order, and we thus did not further consider the effect of orders for the fMRI analysis. Importantly for the fMRI analyses, we also did not measure significant differences in the number of shocks delivered to the victims across our three commander conditions, which simplifies the interpretation of the fMRI data. The number of shocks given also did not differ significantly between the current experiment and the agent experiment reported in Caspar et al. (2020b; HumanAgent comparison: 95% CI = −0.995–0.091; *t*_(53)_ = −1.661; *p *=* *0.103; Cohen’s *d* = −0.454; BF_10_ = 0.851; RobotAgent comparison: 95% CI = −1.059; *t*_(53)_ = −1.892; *p *=* *0.064; Cohen’s *d* = −0.517; BF_10_ = 1.183).

## Materials and Methods: Study 2

### Participants

Forty-eight participants (24 males, 24 females) were recruited in 24 dyads. None of the participants reported knowing each other. The mean age was 23.90 (SD = 3.93). We recruited a larger sample than in Study 1 because we expected to have to reject more participants because the testing took place in a month of the year involving very hot temperatures and the EEG data were particularly difficult to acquire because of sweat artifacts. The following exclusion criteria were determined before further analysis: (1) failure to understand the task; (2) failure to perform correctly the task measuring the implicit sense of agency; and (3) failure to obtain a good signal-to-noise ratio for EEG recordings. To identify participants for whom the estimated action–tone intervals did not gradually increase with the real action–tone intervals, we performed Pearson correlations. When the Pearson *r* value was <0.1, we excluded the action–tone intervals for the corresponding participant. We also did not analyze the action–tone interval data of participants who sent <5 of 60 shocks or >55 of 60 shocks in at least one of the experimental conditions since it would lead to unreliable statistical comparison between Shock and No-Shock trials. Accordingly, the action–tone intervals of 7 of 48 participants were lost because of a Pearson *r* value of <0.1. Fourteen of forty-eight participants sent <5 of 60 shocks or >55 of 60 shocks in at least one of the experimental conditions and their action–tone interval data were lost. The action–tone interval data of one participant was included in the two categories (i.e., *r* < 0.1; an unreliable number of shock–no-shock trials). As a result, we lost the action–tone interval data of 20 of 48 participants, but their other data were kept. The EEG data of 18 participants were not analyzed: 3 because of too many visual artifacts, head artifacts, and/or sweat artifacts; and 15 because they delivered only a small number of shocks (<5 of 60; *N* = 11) or a high number of shocks (>55; *N* = 4) in either one or all conditions. This would indeed prevent obtaining a reliable difference between Shock and No-Shock trials (Caspar et al., 2020b). Thus, we had 28 participants included in the interval estimation task and 30 participants included in the EEG data. The study was approved by the local ethics committee of the Université libre de Bruxelles (reference 018/2015). Data are made available on OSF (https://osf.io/scw9z/).

### Method and material

The method was globally similar to that in Study 1, including the same conditions (Fig. 1*A*) but with a slightly different timing of trials. Each trial started with a fixation cross lasting between 1 and 2 s. When the fixation cross disappeared, participants received a verbal instruction from the experimenter, similar to Study 1. Then, they had to press one of two buttons: SHOCK or NO SHOCK to send an order to an agent, either human or robot. After the agent pressed the button corresponding to the order of the participant, a tone was presented (400 Hz, 200 ms). The interval between the agent’s key press and the start of the beep was 200, 500, or 800 ms. Participants were asked to estimate the elapsed time between their own key press when they sent the order and the beep onset. If a shock was sent to the victim, the shock was delivered at the exact same time as the tone to avoid temporal bias. After 2 s, an analog scale with 0 on the left side and 1500 on the right side of the scale was then displayed on the screen. A red position marker was displayed on that scale with a number, corresponding to the current position of the marker in milliseconds. The starting position of the marker varied randomly on a trialwise basis, and participants were told to ignore the starting position of the marker to provide their final answer. Participants could move the position of the marker along the analog scale by using the same two buttons as for Shock and No-Shock. The keys below the middle fingers allowed them to modify the number associated with the position of the rectangle by steps of ±100 ms. The keys below the index fingers allowed them to modify the answer by steps of ±1 ms. After a fixed duration of 6 s, their answer was saved, and the next trial started. Each participant started with a training session to practice the time interval procedure. The training session lasted for a minimum of eight trials and was repeated until participants declared that they could perform the task correctly.

As in Study 1, to further encourage participants to pay attention to the victim’s hand, on 12 trials in each condition a pain rating scale appeared, ranging from not painful at all (0) to very painful (1000). Participants were asked to rate the intensity of the shock (or no sock) seen on the last trial by moving the red marker bar along the scale using four buttons. The keys below the middle fingers allowed modification of the number associated with the position of the marker by steps of ±100. The keys below the index fingers allowed modification of the answer by steps of ±1. After a fixed duration of 6 s, their answer was saved, and the next trial started. If no shocks were delivered on that trial, participants were asked to report that the shock was not painful at all.

To preserve the same experimental setup between Study 1 and Study 2, participants were isolated in a room and victims were in another room with the camera displaying their hand in real time on the participant’s screen. In all three experimental conditions, the experimenter came to talk to the participant before the start of each experimental condition but then left the room by mentioning that it was to avoid too many interferences in the EEG recordings because of her presence. Participants were told that they would hear the experimenter’s instructions through the headphones.

Each experimental condition was composed of 60 trials. Order of the experimental conditions was counterbalanced across participants. The same questionnaires as in Study 1 were presented to participants at the end of the experimental session.

### EEG recordings

Brain activity was recorded using a 64-channel electrode cap with the ActiveTwo system (BioSemi), and data were analyzed using Fieldtrip software (Oostenveld et al., 2011). The activities from left and right mastoids and from horizontal and vertical eye movements were also recorded. Amplified voltages were sampled at 2048 Hz. Data were referenced to the average signal of the mastoids and filtered (low-pass filter at 50 Hz; high-pass filter at 0.01 Hz). Artifacts because of eye movements were removed based on a visual inspection with the removal of epochs containing eye blinks or ocular saccades. Because of the EEG recordings, participants were further instructed to wait a minimum of 1 s in a relaxed position before pressing a key, so as to obtain a consistent and noise-free baseline taken −500 to −300 ms before the occurrence of the tone. Participants were additionally instructed not to move for up to 2 s after the tone and asked to avoid blinking when they pressed a button. To ensure that participants respected the 2 s without moving and blinking after the tone, they were told to wait for the timescale to appear on the screen. Trials in which participants disobeyed the orders of the experimenter were removed from the analysis.

**Figure 6.**
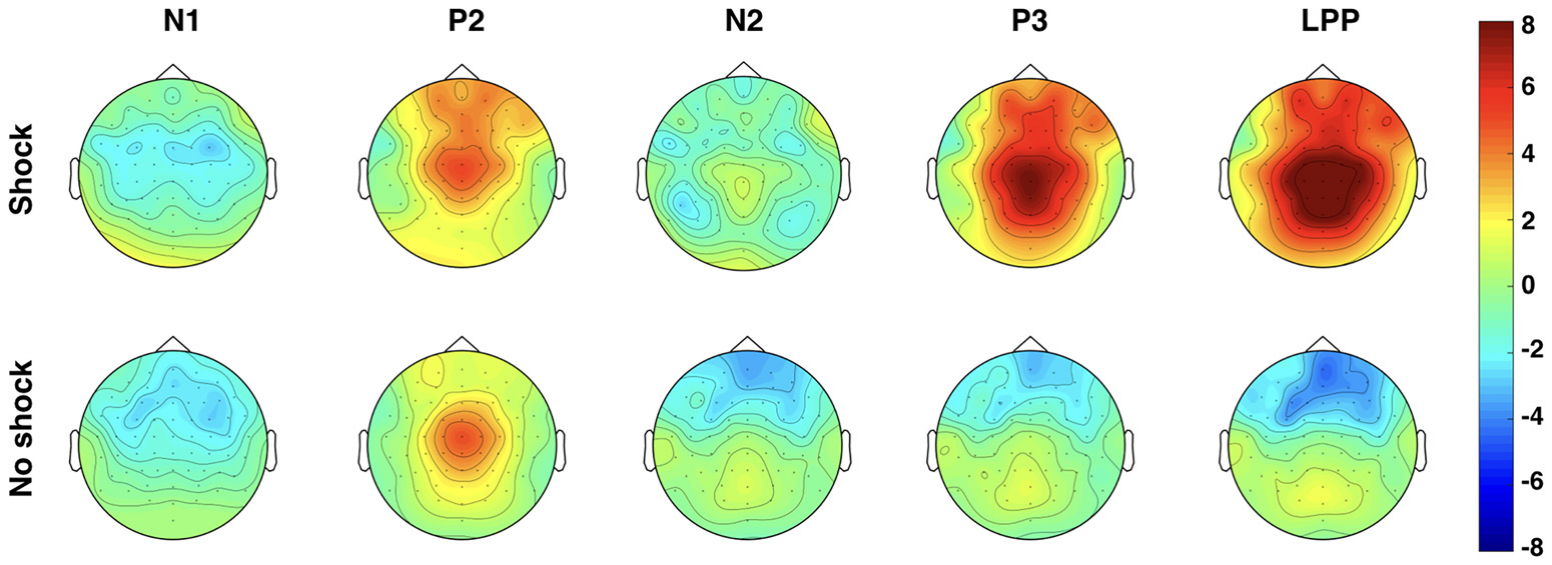
Topographical distributions in Shock and No-Shock trials for the N1, P2, N2, P3, and LPP ERPs.

All event-related potentials (ERPs) were analyzed across Fz, FCz, Cz, CPz, and Pz, similar to past studies (Fan et al., 2013; Cheng et al., 2014) and confirmed with the topographical distributions (Fig. 6). The ERP components were chosen according to visual inspection of the grand-averaged data similar to past studies (Fan et al., 2013; Chen et al., 2014; Cheng et al., 2014) as well as prior knowledge based on a meta-analysis (Coll, 2018). The N1 and the N2 were measured as the most negative peaks within the 30–130 ms time window and the 240–340 ms time window after the tone, respectively. The P2 and the P3 were measured as the most positive peaks within the 130–230 ms time window and the 340–440 ms time window after the tone, respectively. The early LPP (eLPP) and the late LPP (lLPP)were measured as the mean amplitude between the 440–650 ms time window and the 650–900 ms time window after the tone, respectively (Fig. 6, display of a topographical representation).

Source reconstruction was conducted on the grand average of EEG data that were computed with a noise covariance estimation. Minimum norm estimation (Dale and Sereno, 1993) was applied to reconstruct the sources of ERP components. The volume construction was based on a standard head model and source model downloaded through Fieldtrip. Having performed the source localization, we used it to create brain maps showing the brain regions involved in the activity associated with each ERP. We performed this operation with custom-written Python software that uses as input the results of our source localization (https://github.com/ldeangelisphys/ft2nii/). The software assigns each localized source to the corresponding voxel in a standard 2 mm MNI template (MNI152), performs a temporal average over the time window corresponding to the ERP of interest, and a spatial smoothing of the resulting map with a 5 mm Gaussian kernel.

## Results: Study 2

### Number of shocks delivered

**Figure 7.**
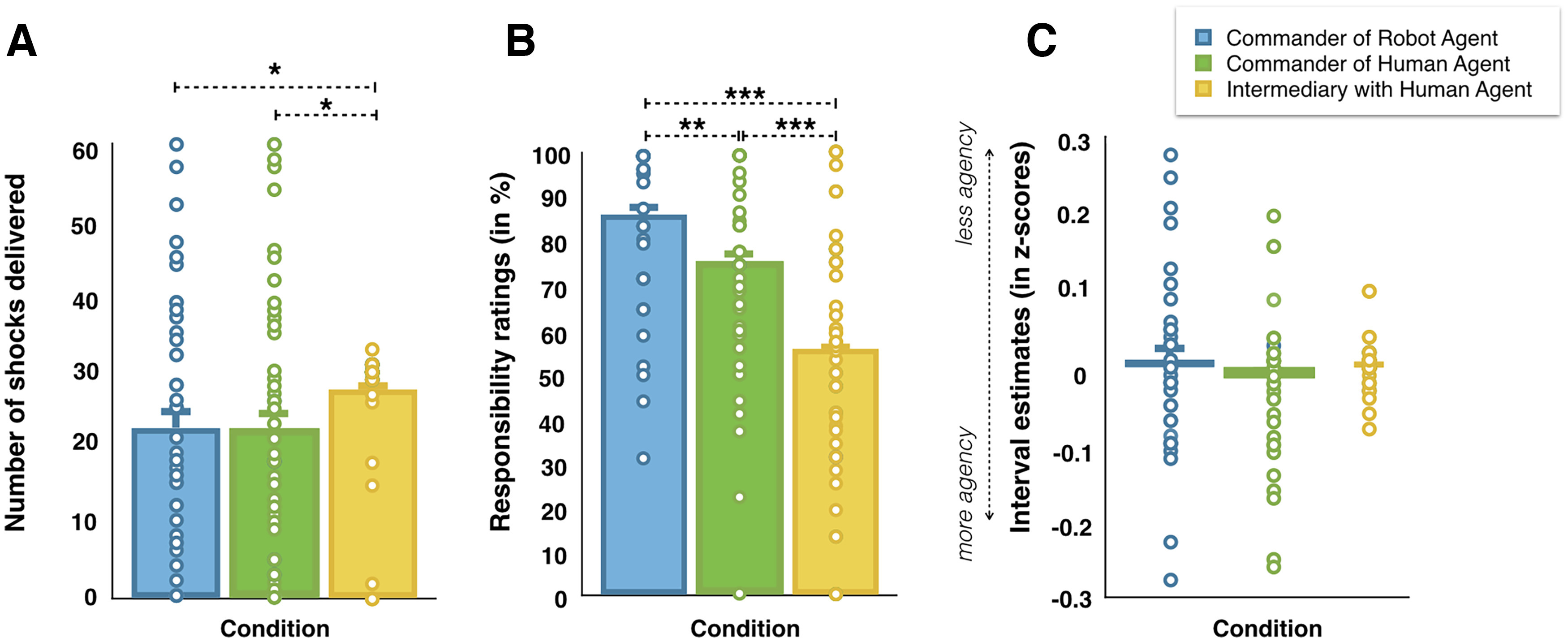
***A***, Graphical representation of the number of shocks delivered in the three experimental conditions. ***B***, Graphical representation of responsibility ratings in the three experimental conditions. ***C***, Graphical representation of *z* scores of interval estimates in the three experimental conditions. All tests were two tailed. ***p ≤ 0.001 and BF_10_ > 3. **p > 0.001 and BF_10_ > 3. *p > 0.01. Errors bars represent standard errors.

In the IntermediaryWithHumanAgent, participants were ordered by the experimenter, on a trial basis, to tell the human agent to inflict 30 of 60 shocks on the victim, randomly. In the commander conditions, participants could freely decide which order to send to the agent. Descriptive statistics indicated that in the CommanderOfHumanAgent condition, participants asked the agent to administer 22.46 of 60 shocks (SD = 17.80; minimum, 0 shocks; maximum, 60 shocks) to the victim. In the CommanderOfRobotAgent condition, participants asked the agent to administer 22.56 of 60 shocks (SD = 17.90; minimum, 0 shocks; maximum, 60 shocks) to the victim. In the IntermediaryWithHumanAgent, participants transmit the order to send a shock to the victim on 27.54 of 60 trials (SD = 7.59; minimum, 0 trials; maximum, 33 trials). Thirteen of 48 participants reported that they voluntarily disobeyed the orders of the experimenter on some trials in the IntermediaryWithHumanAgent condition. Among those 13 participants, 10 disobeyed prosocially, that is, by refusing to send a shock during shock trials and by telling the agent not to deliver a shock to the victim even if the experimenter asked them to do so (i.e., prosocial disobedience), and 3 disobeyed by contradiction; that is, they disobeyed as often on don’t shock trials and on shock trials. We conducted a repeated-measures ANOVA with Condition (CommanderOfHumanAgent, CommanderOfRobotAgent, and IntermediaryWithHumanAgent) as the within-subject factor and Role Order (commander first, victim first) as the between-subject factor on the number of shocks sent in each experimental condition. We observed a significant main effect of Condition (*F*_(2,92)_ = 4.119; *p *=* *0.019; η^2^_partial_ = 0.082; BF_incl_ = 1.472; Fig. 7*A*). Paired comparisons indicated that participants administered fewer shocks in the CommanderOfHumanAgent condition and in the CommanderOfRobotAgent condition than in the IntermediaryWithHumanAgent condition (*t*_(47)_ = 2.130, *p *=* *0.038, Cohen’s *d* = 0.307; and *t*_(47)_ = 2.051, *p *=* *0.046, Cohen’s *d* = 0.296, respectively). Both the frequentist and the Bayesian results indicated that Role Order was slightly in favor of *H*_0_ (*p *>* *0.3; BF_incl_ = 0.391). The interaction Condition × Role Order was in favor of *H*_0_ (*p *>* *0.2; BF_incl_ = 0.280).

We again performed Levene’s tests to test the equality of variance between each experimental condition. Results indicated that variability was reduced in the IntermediaryWithHumanAgent condition compared with the CommanderOfRobotAgent condition (*F*_(1,94)_ = 42.918; *p *<* *0.001) and compared with the CommanderOfHumanAgent condition (*F*_(1,94)_ = 35.920; *p *<* *0.001). The CommanderOfRobotAgent and the CommanderOfHumanAgent conditions did not differ (*p *>* *0.8).

### Pain scale

Of 48 participants, we had a full dataset for 30 participants. We conducted a repeated-measures ANOVA with Condition (CommanderOfHumanAgent, CommanderOfRobotAgent, and IntermediaryWithHumanAgent) and Pain (Shock, No-Shock) as within-subject factors on the pain ratings. The main effect of Pain was strongly in favor of *H*_1_ (*F*_(2,58)_ = 98.531; *p *<* *0.001; η^2^_partial_ = 0.773; BF_incl_ = ∞), with higher pain ratings when a shock was delivered (399 shocks; 95% CI = 348–451) compared with when no shocks were delivered (54; 95% CI = 2.4–105). The main effect of condition (*p *>* *0.1; BF_incl_ = 0.081) and the interaction (*p *>* *0.7; BF_incl_ = 0.042) were in favor of *H*_0_.

### Responsibility ratings

At the end of each experimental condition, participants had to report how responsible they felt for the outcome of their orders. We conducted a repeated-measures ANOVA with Condition (CommanderOfHumanAgent, CommanderOfRobotAgent, and IntermediaryWithHumanAgent) as the within-subject factor and Role Order (commander first, victim first) as the between-subject factor on the responsibility ratings. Both the frequentist and the Bayesian results supported a main effect of Condition (*F*_(2,92)_ = 28.917; *p *<* *0.001; η^2^_partial_ = 0.386; BF_incl_ = 1.234E + 8; Fig. 7*B*). Paired comparisons indicated that responsibility ratings were higher in the CommanderOfRobotAgent condition (86.2%; 95% CI = 80.9–91.5) than in the CommanderOfHumanAgent condition (75.6%; 95% CI = 68.7–82.5; *t*_(47)_ = 3.280; *p *=* *0.002; Cohen’s *d* = 0.473; BF_10_ = 15.87) and in the IntermediaryWithHumanAgent condition (55.8%; 95% CI = 47.6–54; *t*_(47)_ = −6.985; *p *<* *0.001; Cohen’s *d* = −1.008; BF_10_ = 1.456E + 6). Responsibility ratings were also higher in the CommanderOfHumanAgent condition than in the IntermediaryWithHumanAgent condition (*t*_(47)_ = −4.538; *p *<* *0.001; Cohen’s *d* = –0.655; BF_10_ = 545). The Role Order (*F*_(1,46)_ = 0.140; *p *>* *0.7; BF_incl_ = 0.208) and the interaction (*F*_(22,92)_ = 0.077; *p *>* *0.9; BF_incl_ = 0.106) were in favor of *H*_0_ and thus did not show evidence for influencing responsibility ratings.

### Sense of agency

Because interval estimates were planned to be correlated with other measurements in future analyses, we first transformed the raw interval estimates in *z* score data. It is indeed known that participants may differ in the way they use the ms-scale to provide an answer, some preferring smaller numbers and others preferring larger numbers (Caspar et al., 2020a; Cravo et al., 2013). The *z* scores reduce irrelevant intersubject variability by subtracting from each interval estimate the mean estimate for that participant across all trials and by dividing the resulting differences by the SD of all estimates for that participant. The *z*-scored interval estimates are interpreted in the same way as the raw interval estimates, with lower *z*-scored interval estimates being interpreted as a higher SoA. Trials where participants disobeyed the orders from the experimenter were removed from the analyses. We conducted a repeated-measures ANOVA with Condition (CommanderOfHumanAgent, CommanderOfRobotAgent, and IntermediaryWithHumanAgent) and Shocks (Shock, No-Shock) as the within-subject factor and Role Order (commander first, victim first) as the between-subject factor on *z* scores. The main effects of Condition and Role Order were in favor of *H*_0_ (*p *>* *0.3, BF_incl_ = 0.036; and *p *>* *0.9, BF_incl_ = 0.115, respectively; Fig. 7*C*). The main effect of Shock was inconclusive (*p *>* *0.1; BF_incl_ = 1.547). All the interactions were in favor of *H*_0_ (all *p* values* *>* *0.1, all BF_incl_ values ≤ 0.199). We further ran exploratory analyses to investigate whether self-reported personality traits influenced the *z*-scored interval estimated when commanding freely or being an intermediary. We thus computed a “commander effect,” which is the difference between the IntermediaryWithHumanAgent condition and the corresponding CommanderOfHumanAgent condition (i.e., IntermediaryWithHumanAgent – CommanderOfHumanAgent). With this subtraction, higher positive values indicated more SoA in the CommanderOfHumanAgent condition than in the IntermediaryWithHumanAgent condition. We observed little evidence that the fewer the number of participants scored on the ASC submission scale, the lower their interval estimates were in the CommanderOfHumanAgent condition compared with the IntermediaryWithHumanAgent condition (*r* = –0.392; *p *=* *0.022; BF_10_ = 2.628). Correlations with the other subscale were inconclusive or in favor of *H*_0_ (all *p* values* *>* *0.042; all BF_10_ values < 1.511).

Regarding the distribution of the data points, we also performed Levene’s tests to test the equality of variance between each experimental condition. Results indicated that variability was reduced in the IntermediaryWithHumanAgent condition compared with the CommanderOfRobotAgent condition (*F*_(1,79)_ = 22.093; *p *<* *0.001) and the CommanderOfHumanAgent condition (*F*_(1,78)_ = 22.109; *p *<* *0.001). The CommanderOfRobotAgent and the CommanderOfHumanAgent conditions did not differ (*p *>* *0.1).

### EEG results

**Figure 8.**
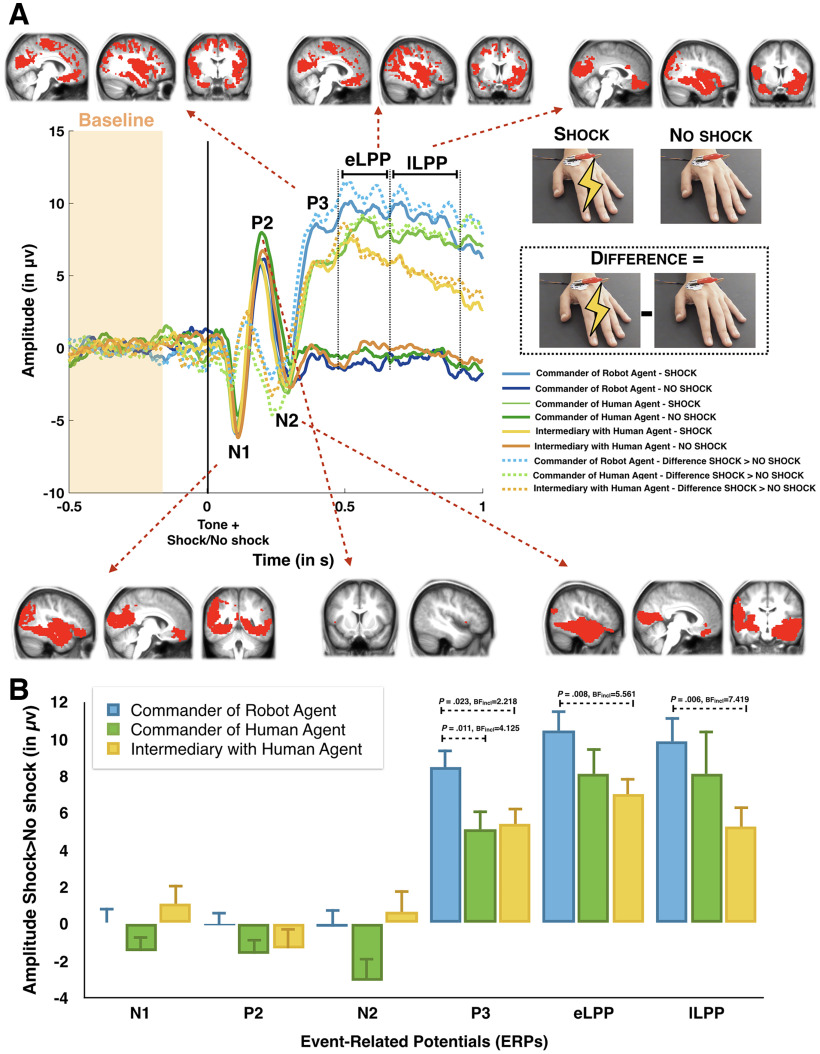
***A***, Grand average ERP for the Shock trials (light full lines) and the No-Shock trials (dark full lines). The dotted lines represent the difference Shock–No-Shock trials. Extended Data Figure 8-1 displays the results over Fz, Cz, and Pz. Source reconstruction maps for each ERP are displayed along each ERP for the difference Shock–No-Shock trials irrespective of condition for the voxels with the highest 5% of positive values. Detailed results from the source reconstruction analyses can be found in Extended Data Figures 8-2 and 8-3. ***B***, Graphical representation of the amplitude in μV of each ERP in each experimental condition. All tests were two tailed. Only significant pairwise comparisons are shown (Table 1; see text for the other comparisons). Source reconstruction maps represent the highest 5% of activation. Errors bars represent standard errors.

We compared the neural processing of pain with an electroencephalogram when participants witnessed a shock being delivered to the hand of the victim. We extracted, based on both previous literature and the visual inspection of the grand averaged waves, the amplitude of several event-related potentials associated either with auditory outcome processing (N1, P2, N2; Luck, 2012) or with pain outcome processing (P3, eLPP, lLPP; for a meta-analysis, see Coll, 2018; Fig. 8*A*).

**Table 1:** Pairwise comparisons of Shock–No-Shock difference across conditions

ERP	Comparisons	Mean (μv)	SD	df	*t*	*p*	*p*FDR	Cohen’s *d*	BF_10_
P3	*Intermed–CH*	*5.4–5.1*	*4.7–5.9*	*30*	*0.278*	*0.783*	*0.783*	*0.050*	*0.199*
	Intermed–CR	5.4–8.5	4.7–5.2	30	−2.396	0.023	0.034	−0.430	2.218
	**CH–CR**	**5.1–8.5**	**5.9–5.2**	**30**	**−2.712**	**0.011**	**0.033**	**−0.487**	**4.125**
eLPP	*Intermed–CH*	*7–8.1*	*5.1–7.7*	*30*	*−0.654*	*0.518*	*0.518*	*−0.117*	*0.233*
	**Intermed–CR**	**7–10.5**	**5.1–5.8**	**30**	**−2.857**	**0.008**	**0.024**	**−0.513**	**5.561**
	CH–CR	8.1–10.5	7.7–5.8	30	−1.888	0.069	0.103	−0.339	0.920
lLPP	*Intermed–CH*	*5.2–8.1*	*6.3–13*	*30*	*−1.059*	*0.298*	*0.436*	*−0.190*	*0.320*
	**Intermed–CR**	**5.2–9.8**	**6.3–7.3**	**30**	**−2.992**	**0.006**	**0.018**	**−0.537**	**7.419**
* *	*CH–CR*	*8.1–9.8*	*13–7.3*	*30*	*−0.790*	*0.436*	*0.436*	*−0.142*	*0.255*

For the three ERPs that are sensitive to shocks, the table summarizes the pairwise comparison across conditions. Mean, Mean voltage (in microvolts) per condition; SD, SD across participants; df, *t*, and *p* summarize the two-tailed Student’s *t* test; BF10, Bayesian equivalent; Intermed, IntermediaryWithHumanAgent condition; CH, CommanderOfHumanAgent condition; CR, CommanderOfRobotAgent condition. Bold type indicates results in favor of *H*_1_ with both the frequentist and the Bayesian approaches; italic type indicates results in favor of *H*_0_ (nonsignificant frequentist statistics and BF_10_ values less than one-third). *p*_FDR_ displays the *p* values after correction with the FDR approach.

10.1523/ENEURO.0508-21.2022.f8-1 Figure 8-1 Tables displaying the paired comparisons for Shock and No-Shock trials on Fz, Cz, and Pz. All tests were two tailed. Download Figure 8-1, DOCX file.

10.1523/ENEURO.0508-21.2022.f8-2 Figure 8-2 Maps derived reconstructing the EEG signal for the contrasts [CommanderOfHumanAgent(S-NS)–IntermediaryWithHumanAgent(S-NS)] in green, [CommanderOfRobotAgent(S-NS)–IntermediaryWithHumanAgent(S-NS)] in cyan, and [CommanderOfRobotAgent(S-NS)–CommanderOfHumanAgent(S-NS)] in violet. The voxels with the highest 5% of values encoding for regions that are the most positively involved in the selected ERP are displayed. Download Figure 8-2, TIF file.

10.1523/ENEURO.0508-21.2022.f8-3 Figure 8-3 To compare Study 1 and Study 2, and to ensure that witnessing a shock versus no shock being delivered to the victim’s hand involve a similar brain activation pattern, we overlaid the MRI results obtained in Study 1 to the EEG results obtained in Study 2 for the Shock–No-Shock contrast. Overlap (in orange) is shown between the maps from the GLM analyses for Shock–No-Shock contrast (in red) and the 5% higher activation map derived reconstructing the EEG signal for the Shock–No-Shock contrasts using the mean activation from components P3, eLPP, and lLPP (in yellow). Download Figure 8-3, TIF file.

We first compared the amplitude of those potentials when participants witnessed a shock on the victim’s hand to when they did not witness that shock to identify the ERPs sensitive to observing pain. To do so, we averaged the amplitude of each ERP across the three experimental conditions for Shock and No-Shock trials. Results supported evidence for a higher amplitude for shock trials compared with No-Shock trials for the P3 (*t*_(30)_ = 10.108; *p *<* *0.001; Cohen’s *d* = 1.815; BF_10_ = 2.673E + 9), the early LPP (*t*_(30)_ = 10.890; *p *<* *0.001; Cohen’s *d* = 1.956; BF_10_ = 1.443E + 9), and the lLPP (*t*_(30)_ = 7.044; *p *<* *0.001; Cohen’s *d* = 1.265; BF_10_ = 187382.55). This difference was in favor of *H*_0_ for the N1 (*p *>* *0.8; BF_10_ = 0.195) and the N2 (*p *>* *0.3; BF_10_ = 0.308), and inconclusive for the P2 (*p *= 0.048; BF_10_ = 1.214). Those results confirmed that the P3, the early LPP, and the late LPP were sensitive to seeing a painful stimulus delivered to the hand of the victim. Of note, these results were also in favor of *H*_1_ for each electrode taken separately on Fz or Cz or Pz (all *p* values* *<* *0.001; all BF_10_ values > 11,713.97; Extended Data Fig. 8-1).

To evaluate how the experimental conditions influenced the neural response to the pain of the victim, we then performed a repeated-measures ANOVA with Condition (CommanderOfHumanAgent, CommanderOfRobotAgent, and IntermediaryWithHumanAgent) as the within-subject factor and Role Order (commander first, victim first) as the between-subject factor on the computed difference between Shock and No-Shock trials for those potentials showing a Shock–No-Shock effect (i.e., P3, early LPP, and the late LPP). For the P3, we observed a main effect of condition (*F*_(2,58)_ = 4.502; *p *=* *0.015; η^2^_partial_ = 0.134; BF_incl_ = 3.0958; Fig. 8*B*). Paired comparisons supported that the amplitude of the P3 was higher in the CommanderOfRobotAgent condition than in the CommanderOfHumanAgent condition (*t*_(30)_ = −2.712; *p *=* *0.011; Cohen’s *d* = −0.487; BF_10_ = 4.125). We also observed that the amplitude of the P3 was higher in the CommanderOfRobotAgent condition than in the IntermediaryWithHumanAgent condition (*t*_(30)_ = −2.396; *p *=* *0.023 Cohen’s *d* = −0.430). But this difference was inconclusive with the Bayesian approach (BF_10_ = 2.218). Table 1 displays the results of the paired comparisons between conditions. We found evidence in favor of *H*_0_ for the main effect of Role Order (*p *>* *0.7; BF_incl_ = 0.251) and for the interaction (*p *>* *0.3; BF_incl_ = 0.264). For the eLPP, the main effect of Condition was significant (*F*_(2,58)_ = 3.465; *p *=* *0.038; η^2^_partial_ = 0.099) but inconclusive with the Bayesian approach (BF_incl_ = 1.197). We found evidence in favor of *H*_0_ for the main effect of Role Order (*p *>* *0.9; BF_incl_ = 0.318) and slightly in favor of *H*_0_ for the interaction (*p *=* *0.09; BF_incl_ = 0.564). For the lLPP, the main effects of Condition and Role Order were inconclusive (*p *>* *0.09, BF_incl_ = 0.709; and *p *>* *0.5, BF_incl_ = 0.428, respectively). The interaction was significant with the frequentist approach (*F*_(2,58)_ = 3.596; *p *=* *0.034; η^2^_partial_ = 0.102) but inconclusive with the Bayesian approach (BF_incl_ = 0.977).

### Relationship between number of shocks freely ordered and temporal binding, feeling of responsibility, and ERP

**Figure 9.**
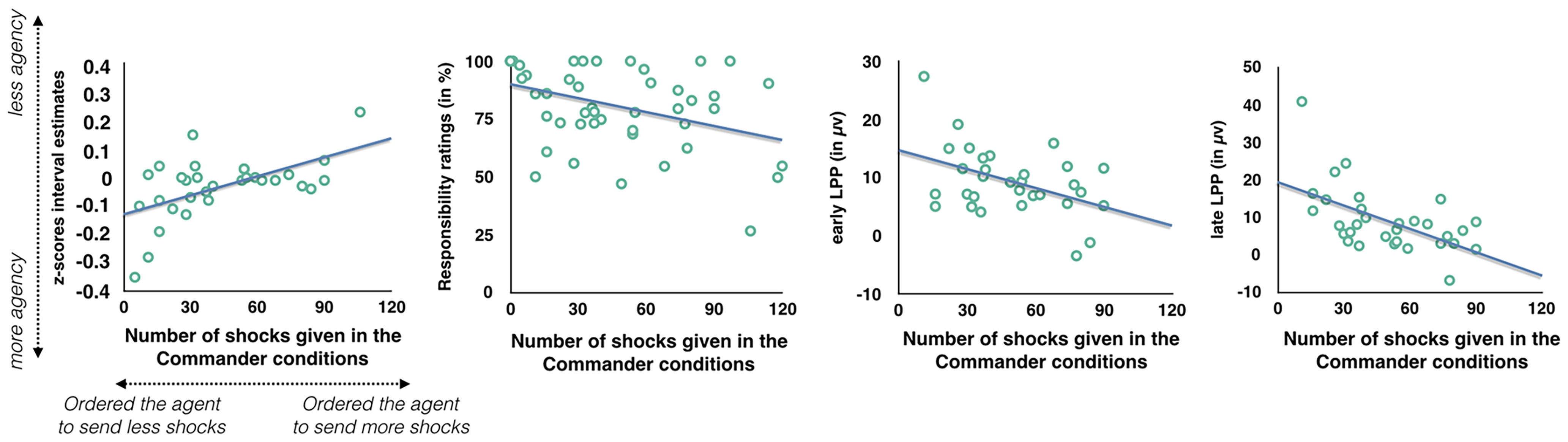
Graphical representations of Pearson correlations between the number of shocks given in the commander conditions and the sense of agency (top left), responsibility ratings (middle left), amplitude of the eLPP (middle right), and amplitude of the lLPP (top right). All tests were two tailed.

To investigate to what extent the sense of agency, empathy for pain, and feeling of responsibility drive prosocial behaviors, we further performed Pearson correlations. To create a single variable, “free-choice condition,” regardless of the type of agent, we summed the data of the CommanderOfHumanAgent and CommanderOfRobotAgent conditions for the number of shocks delivered. We then computed an average score across the same conditions for the *z* scores of interval estimates, used as a proxy for the sense of agency, for responsibility ratings, and for ERPs that were sensitive to the victim’s pain (i.e., P3, eLPP, and lLPP). For these ERPs, we computed a general pain response by subtracting the amplitude of those potentials during No-Shock trials from Shock trials (i.e., Shock–No-Shock). To correct for multiple comparisons with the frequentist statistics, we applied a false discovery rate (FDR) approach with the Benjamin and Hochberg (1995) method to each *p*-value. Both frequentist and Bayesian statistics for those correlations were two tailed. We observed evidence that the number of shocks freely administered to the victim correlated positively with the *z* scores of interval estimates (*r* = 0.443; *p*_FDR_ = 0.022; BF_10_ = 3.311), indicating that the higher were the *z* scores, which is interpreted as a reduced sense of agency, the higher was the number of shocks sent to the victim (Fig. 9). We also observed evidence for a negative correlation between the number of shocks freely delivered and responsibility ratings (*r* = –0.393; *p*_FDR_ = 0.015; BF_10_ = 7.372). This suggests that the more responsible the participants felt, the fewer shocks they sent to the victim. Correlations with ERPs revealed evidence for a negative correlation of the number of shocks ordered with the late LPP (*r* = –0.561; *p*_FDR_ = 0.005; BF_10_ = 38.575) and the early LPP Shock–No-Shock magnitudes (*r* = –0.434; *p*_FDR_ = 0.022; BF_10_ = 3.857), suggesting that the higher were the Shock–No-Shock amplitudes of the early and late LPPs, the lower was the number of shocks delivered. Of note, these results stayed similar when considering only the first half or the second half of the trials, thus controlling for the repetition suppression effect. The Bayesian approach indicated that the correlation between the number of shocks and P3 was slightly in favor of *H*_0_ (*p*_FDR_ > 0.2; BF_10_ = 0.384). Of note, the number of shocks delivered freely to the victim did not correlate with the amplitude of ERPs, which were not found to be sensitive to the pain of the victim (i.e., N1, P2, N2: all *p*_FDR_ values > 0.072; BF_10_ values ≥0.609 and ≤1.924).

Taken separately, the same correlations gave similar results in favor of *H*_1_ in the CommanderOfRobotAgent condition. However, the same correlations, although going in the same direction, were inconclusive with the Bayesian approach in the CommanderOfHumanAgent condition (all *p* values* *>* *0.041; all BF_10_ values >0.591 and <1.658).

### Studies 1 and 2: self-report personality questionnaires

We conducted correlational, exploratory analyses on both studies combined to investigate self-reported personality traits associated with the number of shocks freely delivered in the commander conditions, regardless of the types of agent. Since the analyses were exploratory, analyses were two tailed. Again, we corrected for multiple comparisons with the FDR. Results revealed that the higher participants scored on the Levenson Primary Psychopathy scale, the more shocks they freely ordered the agent to send to the victim (*r* = 0.370; *p*_FDR_ = 0.001; BF_10_ = 61.170). We also observed evidence for two positive correlations between the number of shocks delivered and the purity (*r* = 0.337; *p*_FDR_ = 0.011; BF_10_ = 20.067) and the authoritarianism (*r* = 0.304; *p*_FDR_ = 0.027; BF_10_ = 7.550) subscales of the Moral Foundation Questionnaires. Other correlations were in favor of *H*_0_ or inconclusive (all BF_10_ values >0.137 and <1.61).

## Discussion: Study 2

In Study 2, in addition to the feeling of responsibility for the outcomes of one’s own action, we also integrated an implicit measure of the sense of agency over one’s own action based on interval estimates. A previous study showed that the sense of agency and the feeling of responsibility can be influenced similarly by obedience to authority and freedom of choice (Caspar et al., 2021), and another study showed that following a training session emphasizing responsibility can enhance the sense of agency (Caspar et al., 2020a). However, they refer to two different phenomenological experiences and may thus also have different relations to behaviors (Balconi, 2010). While the sense of agency refers to the feeling of authorship over an action, the feeling of responsibility relates rather to the processing of the outcome of this action.

We did not observe statistical differences in interval estimates, which were used as a proxy for the sense of agency among the three experimental conditions. This suggests that the sense of agency (unlike the sense of responsibility) does not differ between commanding and being a mere intermediary. However, with the present results we cannot argue in favor of an equally low sense of agency when commanding or being a mere intermediary, or in favor of a high sense of agency for both positions in the command chain. A control condition, in which participants are the direct agent of the action, would have allowed understand whether or not the sense of agency is reduced when people give orders to a third party. Yet, in a former study in which participants took the role of either the agent or the commander in a within-subject design (Caspar et al., 2018), results indicated that commanding an agent led to a reduction of the sense of agency, also measured with the method of interval estimates. We further observed that self-reported personality traits modulated this effect, with participants scoring higher on the ASC scale having the lower commander effect.

An interesting finding indicates that although the means of interval estimates did not differ among the three experimental conditions, variance was strongly reduced in the IntermediaryWithHumanAgent condition compared with the two commander conditions. This may suggest that when people simply transmit the orders of another individual, a higher conformity at the cognitive level is observed compared with freely deciding.

While several scientific publications highlighted the role of the sense of agency in prosocial attitudes (Gallagher et al., 2012; Haggard, 2017), direct correlations between interval estimates and prosocial behaviors had barely been shown. Here, we observed a correlation between *z*-scored interval estimates and prosocial behaviors in the commander conditions, regardless of the type of the agent (i.e., robot or human). This suggests that participants with a high sense of agency when sending orders to an agent also tend to act more prosocially by avoiding inflicting pain on the victim too frequently. Results further showed that participants who experienced a higher feeling of responsibility when commanding an agent also sent fewer shocks to the victim. This highlights the role of experiencing oneself as the author of an action and feeling responsible for its outcomes in prosocial decision-making.

In the study byCaspar et al. (2020b), agents rated as less painful the pain stimulations that they delivered to the victim in the coerced condition compared with when they were free to decide. Driven from these results, we performed these analyses in the present study, but there were no differences in the pain ratings across the experimental conditions. Having a main effect of shock nonetheless confirmed that participants were perceiving the painfulness of the simulations.

Regarding our experimental manipulations, we observed that participants had a higher neural response when they could command a robot compared with when they could command a human. Since participants can diffuse more of their responsibility toward a human agent than toward a nonhumanoid robot agent (because the latter is perceived as less intentional; Wang and Quadflieg, 2015; Sahaï et al., 2022), this result supports the idea that the process of displacement of responsibility plays a key role in pain processing. With the frequentist approach, we also observed that participants had a higher neural response when they could command a robot compared with when they were intermediary with a human. Together, those results could suggest that when they are in the role of the commander, participants have a higher neural response to the pain of the victim when they cannot displace their responsibility toward another human.

Interestingly, the difference between commanding a human and commanding a robot was observed for the P3, but not on the eLPP and the lLPP. However, commanding a robot and being an intermediary with a human agent involved a greater difference for the eLPP and for the lLPP, but not for the P3. The literature on the P3 and the LPP does not offer a concrete conceptual distinction between these two components, with some authors arguing that the LPP is a simple extension of the P3 (Olofsson et al., 2008). However, together, these results suggest that the P3 and the LPP could be influenced by different social factors. This would be consistent with another former study manipulating social power (Galang et al., 2021).

The N1 and N2 amplitudes did not statistically differ between painful and nonpainful trials. A possibility is that the N1 and the N2 components also partially reflect the neural processing of auditory outcomes (i.e., tones) on centroparietal sites (Luck and Kappenman, 2011), for which we did not expect a difference for painful and nonpainful stimuli. However, as (Coll, 2018) indicated in a recent meta-analysis, even former studies that indicated a difference between the amplitude of the N2 between painful and nonpainful stimuli, N1 and N2 are not reliably associated with vicarious pain observation.

Interestingly, while the P3 appeared to be more influenced than the LPP by social power, the LPP correlated with prosocial behaviors while the P3 did not. We indeed observed that the higher the amplitudes of the early and late LPPs, the fewer shocks participants ordered to send to the victim. This is in line with former studies that showed that a higher neural empathic response leads to more prosocial attitudes toward others (Gallo et al., 2018).

In accordance with former studies (for a meta-analysis, see Coll, 2018), we observed that P3, especially, but also the early and late LPPs were sensitive to the observation of the painful shock delivered to the victim’s hand. Importantly, the difference observed between Shock and No-Shock trials could also be attributed to differences in movement across the two conditions: participants saw a muscular twitch in Shock trials but not in the No-Shock trials. Thus, we ran an additional control study (i.e., Study 3) in which participants in the role of the commander could send either a painful shock to the victim or a nonpainful shock.

## Materials and Methods: Study 3

### Participants

Thirty-one participants (10 males, 21 females) were recruited in 16 dyads. None of the participants reported knowing each other. Of note, one participant failed to present himself on the agreed time at the laboratory, and the participant that would have been paired with him therefore only played the role of the commander. The mean age was 22.26 years (SD = 2.9). The following exclusion criteria were determined before further analysis: (1) failure to understand the task; or (2) failure to obtain a good signal-to-noise ratio for EEG recordings. The EEG data of 10 participants were not analyzed: 4 because of too many visual artifacts, head artifacts, and/or sweat artifacts; and 6 because they delivered too small a number of shocks (<5 of 60 shocks; *N* = 4) or too high a number of shocks (>55 shocks; *N* = 2) in either one or all conditions. This would have prevented estimating reliable difference between painful shock and nonpainful shock trials. The study was approved by the local ethics committee of the Université libre de Bruxelles (reference 018/2015). Data are made available on OSF (https://osf.io/scw9z/).

### Method and procedure

To preserve the same experimental setup as in Study 2, participants were isolated in a room and victims were in another room with the camera displaying their two hands in real time on the participants’ screens. There were two experimental conditions: a CommanderOfHumanRobot condition, where participants could decide which order to give to a robot executing their order; and an IntermediaryWithRobotAgent condition, where participants transmitted the order received by the experimenter to the robot executing their order. In the two experimental conditions, the experimenter came to talk to the participant before the start of each experimental condition but then left the room by mentioning that it was to avoid too many interferences in the EEG recordings because of her presence. Participants were told that they would hear the experimenter’s instructions through the headphones.

**Figure 10.**
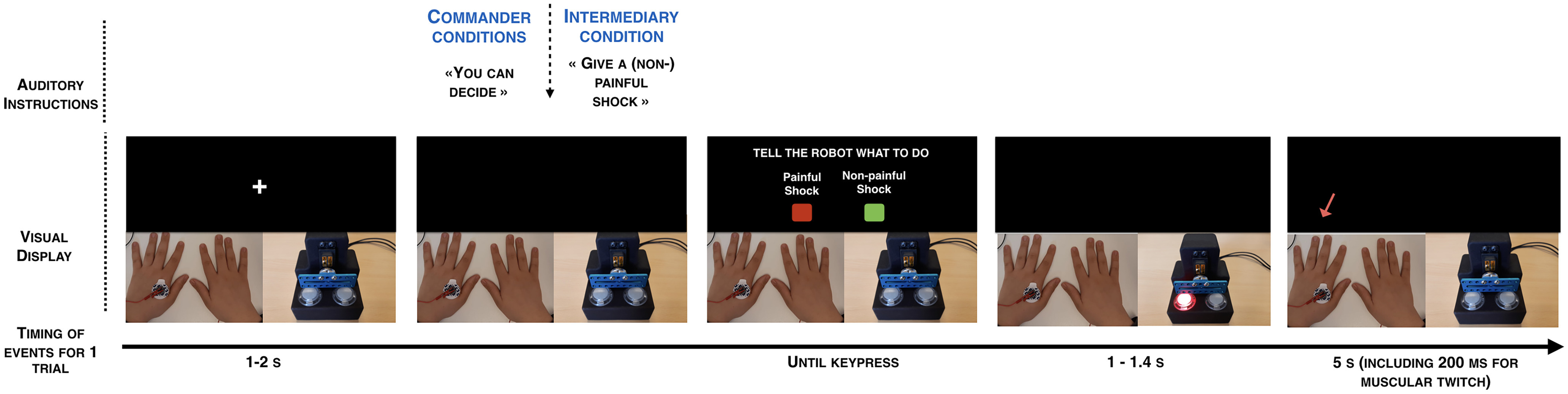
Visual display of the structure of a single trial. Participants in either condition were seeing the two hands of the victim on the bottom left and the robot executing their order on the bottom right. If participants pressed the red or the green button, the corresponding color appeared on the button box of the robot, which then executed the order. A red or green arrow pointing to the hand where the shock was going to be delivered was then displayed on the screen to ensure that participants looked at the correct hand. The only difference between the two experimental conditions (commander vs intermediary) was the auditory instruction they were given at the start of each trial. In the commander condition, they were told “you can decide,” and participants could thus choose freely between pressing the red or green button; in the intermediary condition, they were instructed to “give a painful shock” or “give a nonpainful shock” depending on the trial, and then typically pressed the requested button.

Each trial started with a fixation cross lasting between 1 and 2 s. When the fixation cross disappeared, participants received a verbal instruction from the experimenter. Then, they had to press one of the two buttons, PAINFUL SHOCK, associated with the red color, or NONPAINFUL SHOCK, associated with the green color, to send an order to the robot agent (Fig. 10). The robot then pressed the corresponding button on its own button box. In this control study, a shock was systematically sent. If the PAINFUL SHOCK button was pressed, the shock was set up to be at the pain threshold. If the NONPAINFUL SHOCK button was pressed, the shock was set up to be nonpainful. Before starting the experiment, we determined the painful and nonpainful thresholds on the two hands of each participant. On the right hand, we positioned one electrode on the top of the hand and one electrode on the bottom of the hand. On the left hand, the two electrodes were positioned on the bottom of the hand, and thus were not visible. It was decided to offer a visual cue to the participants, so that they could more easily remember where the painful electrodes were and where the nonpainful electrodes were. An example video can be found on OSF (https://osf.io/scw9z/). For the left hand, we calibrated a threshold to produce a visible muscular twitch for each shock received, but at a nonpainful threshold. To do so, we asked participants to tell us when their fingers were moving but the shock remained not painful. For the right hand, we increased the stimulation up to the pain threshold, similar to Studies 1 and 2. The two hands of the victims were placed below the camera that the commander could see. We kept the electrodes visible on the left hand to remind participants that this corresponded to the painful shock. In addition, when participants pressed the PAINFUL SHOCK or the NONPAINFUL SHOCK button, an arrow, either red or green depending on the key press, appeared and pointed toward the hand that was going to receive the shock. This procedure ensured that participants were actually paying attention to the correct hand at the moment of the shock. Compared with Studies 1 and 2, there was no random mapping of the key press as we could not switch the hand receiving the painful shock and nonpainful shock on each trial. In that study, we did not ask participants to estimate the delay between the key press and the shock as it was not necessary for the control.

Each experimental condition was composed of 60 trials. The order of the experimental conditions was counterbalanced across participants. At the end of the experiment, participants had to fill in a questionnaire assessing how sorry and how bad they felt during each experimental condition.

### EEG recordings

Brain activity was recorded using a 32-channel electrode cap with the ActiveTwo system (BioSemi), and data were analyzed using Fieldtrip software (Oostenveld et al., 2011). The same preprocessing steps and analyses as in Study 2 were performed.

## Results: Study 3

We first compared painful shock trials to nonpainful shock trials to ensure that our ERPs were sensitive to pain processing even if movement was visible in both pain and no-pain trials. The same ERPs as in Study 2 were extracted (i.e., N1, P2, N2, P3, eLPP, lLPP). One participant was excluded because the data deviated >2 SDs. We conducted paired-sample *t* tests between the amplitude of painful shocks and the amplitude of nonpainful shocks in the two experimental conditions combined. The frequentist approach and the Bayesian approach confirmed that the P3 (*t*_(19)_ = 4.302; *p *<* *0.001; Cohen’s *d* = 0.962; BF_10_ = 84.437), the eLPP (*t*_(19)_ = 4.000; *p *<* *0.001; Cohen’s *d* = 0.894; BF_10_ = 46.119), and the lLPP (*t*_(19)_ = 4.495; *p *<* *0.001; Cohen’s *d* = 1.005; BF_10_ = 124.241) differentiated pain from no-pain trials, with a higher amplitude of these ERPs when participant witnessed the painful shocks compared with the nonpainful shocks. Other comparisons were in favor of *H*_0_ (all *p* values* *>* *0.4; all BF_10_ values < 0.290).

**Figure 11.**
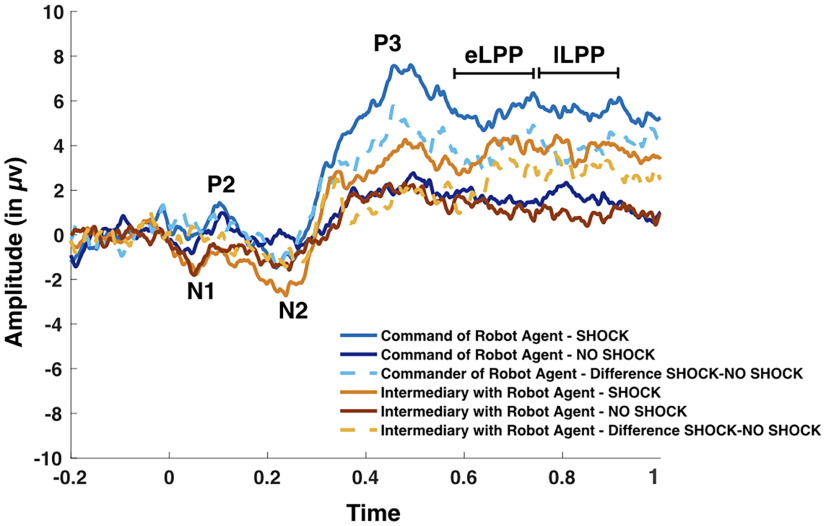
Event-related potentials in painful shock (full lines) and nonpainful shock (dotted lines) in the CommandRobotAgent condition (blue lines) and the IntermediaryWithRobotAgent condition (orange lines).

A putative pain response, corresponding to the subtraction of nonpainful shocks from painful shocks trials was then computed on the P3, the eLPP, and the lLPP to compare command and intermediary conditions and to examine whether we can replicate that command conditions trigger larger pain-related signals than intermediary conditions. We conducted a repeated-measures ANOVA with Condition (CommandRobotAgent and IntermediaryWithRobotAgent) as the within-subject factor on the pain response of the P3, the eLPP, and the lLPP. For the P3, we observed evidence in favor of *H*_1_ for a main effect of condition (*F*_(1,19)_ = 6.749; *p *=* *0.018; η^2^_partial_ = 0.262; BF_incl_ = 3.490), with a higher amplitude of the P3 in the CommandRobotAgent condition (4.2 μv; 95% CI = 2.49–5.90) compared with the IntermediaryWithRobotAgent condition (2 μv; 95% CI = 0.3–3.71; Fig. 11). The same effect of Condition was inconclusive for the eLPP (*p *=* *0.092; BF_incl_ = 1.020) and the lLPP (*p *>* *0.2; BF_incl_ = 0.568).

## Discussion: Study 3

In Study 3, the nonpainful shock triggered a visible muscle twitch, but this twitch was not painful. Results indicated that painful shocks produced a higher amplitude of the P3, eLPP, and lLPP compared with nonpainful shocks, even if both produced a visible movement, thus speaking against the notion that the difference in visible movements in the No-Shock condition suffices to explain the differences in these ERPs in the Shock versus No-Shock contrast. Together, those results suggest that our ERPs reliably reflect pain processing.

We observed that commanding a robot agent led to a greater amplitude of the P3 compared with being an intermediary with a robot agent. This is interesting because in Study 2, the same comparison but with a human agent did not lead to any significant differences. Overall, it would suggest that when people can displace their responsibility toward another human, the difference in social power between the two experimental conditions is not processed as such. However, when people cannot fully displace their responsibility as they give orders to a nonintentional entity, they appear to process the consequences of their orders more (here, the pain inflicted to the victim) when they have more social power. The present study should thus be seen as a first step on a more complex journey toward a better understanding of how commanding robots may influence our cognition.

## General discussion

Past scientific research has shown that being an intermediary in a command chain was associated with a higher prevalence to accept immoral orders (Milgram, 1974). In the present study, our aim was to understand how two different neurocognitive processes, the sense of agency and empathy for pain, differ between being the commander or a simple intermediary.

In a former fMRI study (Caspar et al., 2020b), we observed that for the agent directly delivering a shock, obeying orders reduced vicarious activations toward a victim’s pain compared with acting freely, suggesting that a reduced decisional power negatively impacted the neural empathic response. This result was also confirmed by another study (Galang et al., 2021), which showed that recalling a low-social power situation did not lead to differences in the neural empathic response between painful and nonpainful pictures, while this difference was significant in a high-social power condition. In the present study, when participants were in the role of commander, they had a total social power as they could decide which order to ask an intermediary to execute. In contrast, when they were in the intermediary position, they had to follow the experimenter’s instructions, thus having a reduced social power.

Interestingly, in Study 1 we observed that vicarious activations toward the victim's pain, as measured using a physical vicarious pain signature, or less directly, using voxelwise differences in regions associated with empathy, did not differ strongly enough to lead to significant differences between the commander position and the intermediary position. However, in Study 2 using electroencephalography, we observed that responses in EEG potentials that discriminate Shock from No-Shock observation were higher for commanders than for intermediaries, but only when commanders were giving orders to a robot. Further, in Study 3, the EEG results indicated a higher neural response to the pain of the victim when participants were commanding a robot compared with when they were intermediaries with a robot. Giving orders to an entity that does not have its own individual responsibility is likely to prevent a diffusion of the responsibility phenomenon (Bandura, 2006). This effect was more reliable over the P3 than over the eLPP and the lLPP, which is consistent with former studies (Galang et al., 2021; Pech and Caspar, 2021). To investigate further which areas mediate this difference, we performed source reconstruction on our EEG data, which revealed the involvement of insula and ACC, as we had initially hypothesized. An obvious explanation for the difference between the MRI and EEG results in the insula and ACC is that corrections for multiple comparisons in MRI required a much stricter statistical criterion (FWE, *p *<* *0.001) compared with the EEG analysis. Our MRI study indeed had a statistical power of 80% only for detecting effects of at least *d* = 0.9. None of the significant effects we observed in the EEG had such large effect sizes. When looking at uncorrected fMRI results for the CommanderOfRobotAgent(S-NS)–IntermediaryWithHumanAgent(S-NS) and CommanderOfRobotAgent(S-NS)–CommanderOfHumanAgent(S-NS), we indeed observed ACC and insula activation when looking at uncorrected results. Another possible explanation of the difference among the MRI results in Study 1, which did not show statistical difference between our experimental conditions, and the EEG results in Study 2 and Study 3, which showed a higher amplitude of the P3 when participants commanded a robot, could be that action–outcome intervals have a shorter delay in EEG than in MRI setups. Indeed, in MRI, the outcome followed the agent’s key press by 3–9 s, while in EEG by 200–800 ms, because of the long intervals that are needed in fMRI task design. Former studies indicated that action–outcome delays impact agency, with longer action–outcome intervals impacting the sense of agency (Humphreys and Buehner, 2010). It could be the case that empathy is also impacted by long action–outcome delays.

The overlap between the MRI results in Study 1 and the source reconstruction result in EEG in Study 2 showed convergences but also differences. We indeed observed the involvement of the ACC and the insula in our EEG data, which is consistent with the MRI results on pain observation in Study 1. However, there were also some areas that were not overlapping. A first critical difference was the auditory tone present after each key press in the EEG study but not in the MRI study. A second one is that in the EEG study, the shock appeared 200, 500, or 800 ms after the key press, thus including a lower separation between the motor response and the pain response compared with the MRI study where we use a minimum interval of 3 s between the key press and the shock. A third difference is that in the EEG study, we also asked our participants to estimate the delay between the key press and the tone, a cognitive task that was not present in the MRI study. Future studies where EEG is used within an MRI on the same participants could reveal a more precise overlapping in this context.

When comparing activations from Study 1 to the activations obtained from another MRI study with the same experimental setup but with participants having the position of the agent (Caspar et al., 2020b), we observed that neural activations in areas including IPL and fusiform gyrus were reduced when individuals are commanding another agent compared with when they are agents themselves. In other words, being free to decide which orders to ask another person to execute leads to a more reduced activation in social cognition-related brain regions than being free to both decide and act. When we compared activation patterns between agents coerced and commanders giving orders freely—thus having a classical hierarchical chain between one giving orders and one obeying orders—the agent had higher brain activation than the commander in empathy-related areas as SII and IPL, suggesting that acting has a higher influence of the neural empathic response than having decisional power. The physical vicarious pain signature results also support this notion. Together, our results suggest that having low social power reduces the neural empathic response. We also observed that not being the author of the action impacts this neural empathic response even more.

We also observed that behavioral results slightly differ between Study 1 and Study 2. In Study 1, the number of shocks delivered to the victim did not statistically change across the three experimental conditions, while in Study 2, agents delivered fewer shocks in the CommanderOfHumanAgent condition and in the CommanderOfRobotAgent condition, compared with the IntermediaryWithAHumanAgent condition. We actually observed that participants tended to disobey the orders of the experimenter in the MRI study slightly more compared with in the EEG study. A possible explanation is that, although participants were performing the task with the experimenter close to them in the MRI scanner, social distance with the experimenter could have been perceived as higher because of the MRI scanner and headphones. Also, we observed that in Study 1 participants reported a higher feeling of responsibility in both the CommanderOfHumanAgent condition and the CommanderOfRobotAgent condition compared with the IntermediaryWithAHumanAgent condition, while in Study 2 participants reported more responsibility in the CommanderOfRobotAgent condition compared with the other two conditions. This latter finding perhaps explain why we also observed a higher neural response to the pain of the other in the CommanderOfRobotAgent condition, as previous studies showed a position relationship between the feeling of responsibility and empathy for pain (Lepron et al., 2015; Cui et al., 2015).

In hierarchical situations, one person decides and orders, and another person executes. Thus, deciding and acting are two different cognitive functions that are split across the brains of two different individuals. The data acquired in the present study combined with the data collected in a former study (Caspar et al., 2020b) suggest that being the commander or the intermediary involved reduced brain activations in empathy-related brain regions for the pain inflicted for the victim compared with being free agents that can decide and act themselves. Our results also suggest that coerced agents or commanders experience a reduced agency over their actions and its consequences. These results show how powerful hierarchical situations can facilitate the commission of actions that harm others, as agency and empathy are split across multiple individuals.

## Synthesis

Reviewing Editor: Macià Buades-Rotger, Universitat de Barcelona Decisions are customarily a result of the Reviewing Editor and the peer reviewers coming together and discussing their recommendations until a consensus is reached. When revisions are invited, a fact-based synthesis statement explaining their decision and outlining what is needed to prepare a revision will be listed below. The following reviewer(s) agreed to reveal their identity: Elisabeth Parés-Pujolràs, Artur Czeszumski.

I'd like to thank the authors for the effort they've made to address all of my previous concerns, especially for conducting the suggested extra study to rule out the possibility that the P3 effects were driven not by pain observation but by differences in visual input. I am now happy to recommend this manuscript for publication, provided that the following minor concerns - especially the ones related to the new study - are addressed.

- I believe the Vicarious Pain Signatures analysis is much more clear now, thanks. When you talk about "loading" of the signature, does that mean the weight?

- I am still not very convinced about the authors justification for the selection of the time-windows for the eLPP/lLPP components. The authors replied that they based their division on the grand-averaged data, but in fact as I pointed out the grand-averaged data do not show any visible deflection at the times they set their latency boundaries (see Figure 8). Since the results for these two components always go in the same direction anyway, it is not particularly problematic for this study, but I think it would generally be more sensible to take a single LPP component, given that there doesn't seem to be an objective reason to do otherwise. If the authors choose to keep the analysis as they are, they should justify their choice of time windows on previous literature or a clear, objectively marked deflection in the signal, because the grand-averages do not show any clear delimitation point.

- The topographies in Figure 6 are very useful, thanks. One of the things they show is that the P2, P3 and LPP are strongest around electrode Cz, although a weaker positivity is also present in more frontal areas. Since the authors show the P3 and LPP results are robust in several electrodes, I don't think further work is required here, but for future studies I would advise against the fronto-posterior averaging procedure they used here, because it could often lead to spurious results due to overlapping signals, or conversely to a lack of power. In this study, for example, it might well be that the lack of significant results in P2 is because the authors are averaging electrodes with almost no signal with those that do contain the peak.

- In the new Study 3, could the authors explain why the position of the electrodes on the left and right hands were different (i.e. on top of and under in the right hand, but only under in the left hand)? Also, these positions seem different to the ones used in study 2 - is there a particular reason for that? Did the two electrode positions elicit similar movements, and are there any videos that could be attached as a supplement?

- In the text it says there was one electrode on top of and one under the right hand, and that an electrode on the left hand was kept visible to remind participants that the left hand is the one that received painful shocks. But was the electrode on top of participants' right hands hidden for some reason? Figure 10 shows no electrode on the right hand.

- I still have a small concern regarding the control used in this study, especially relating to the nature of the painful vs. non painful movement. P3-like signals have been related to decision-making, and it is well known that the extent/speed of visual input can have a strong impact on the signal. Thus, if the elicited twitch was a lot smaller in the non-pain compared to the pain conditions, that could still account for the results. If movements were similar in effectors and extent, as the authors state, it should not be the case. Is there any way the authors could measure the movement duration and extent from the participants' hand videos? Adding sample videos as supplementary material to show that the movements are comparable should suffice.

-The general discussion still talks only about study 2, without mentioning the fact that the results there could be driven by a difference in visual stimulation. Please extend the discussion to reflect the results from control study 3.
