# Supplementary material for: Commanding or Being a Simple Intermediary: How Does It Affect Moral Behavior and Related Brain Mechanisms?
Source: eNeuro. 2022 Oct 14;9(5):ENEURO.0508-21.2022. doi: 10.1523/ENEURO.0508-21.2022 (PMC9581580; doi:10.1523/ENEURO.0508-21.2022)
Supplement: Figure 3-1 — BOLD activity of Shock–No-Shock contrast for all experimental conditions together. Only clusters surviving a 5% FWE correction at the cluster size are reported (t = 3.5; p < 0.001; cluster size 160). Brain regions are identified using the Anatomy Toolbox (Eickhoff et al., 2005). Download Figure 3-1, DOCX file. [file enu-eN-CFN-0508-21-s04.docx]

**Extended Data Figure 3-1. BOLD activity of Shocks - Noshock contrast for all experimental conditions together.** Only clusters surviving a 5% FWE correction at the cluster size are reported (t=3.5, p < .001, cluster size 160). Brain regions are identified using the Anatomy Toolbox [(Eickhoff et al., 2005)](https://www.zotero.org/google-docs/?aAGuWi).

| **Cluster size** | **Voxels in cyto** | **% Cluster** | **Hem** | **Cyto or Anatomical description** | **% Area** | **Peak**  **t-value** | **MNI coordinates** | | | |
| --- | --- | --- | --- | --- | --- | --- | --- | --- | --- | --- |
|  |  |  |  |  |  |  | **x** | **y** | **z** | |
| **Shocks - Noshock all experimental conditions**  (5% FWE correction . t=3.5 . p<.001 . k=160) | | | | | | | | | | |
| 3622 | 522.9 | 14.4 | L | Area hOc4la  Middle Temporal Gyrus | 61.2 | 10.49 | -54 | -70 | | 2 |
|  | 460.6 | 12.7 | L | Area hOc4lp  Middle Occipital Gyrus | 53.8 | 5.98 | -28 | -92 | | 12 |
|  | 262.5 | 7.2 | L | Area hOc4v [V4(v)]  Inferior Occipital Gyrus | 36.1 | 7.07 | -30 | -82 | | -6 |
|  |  |  | L | Area hOc4v [V4(v)]  Lingual Gyrus |  | 5.71 | -32 | -86 | | -14 |
|  | 231.1 | 6.4 | L | Lobule VIIa crusI  Cerebelum | 7.6 | 5.11 | -26 | -66 | | -32 |
|  | 192.8 | 5.3 | L | Area FG2  Fusiform Gyrus | 37.8 | 8.04 | -42 | -68 | | -14 |
|  | 183.4 | 5.1 | L | Area FG3  Inferior Temporal Gyrus | 22.2 | 6.30 | -40 | -42 | | -18 |
|  |  |  | L | Area FG3  Fusiform Gyrus |  | 5.88 | -40 | -54 | | -20 |
|  | 143.4 | 4 | L | Area hOc3v [V3v] | 15.5 |  |  |  | |  |
|  | 131.3 | 3.6 | L | Lobule VI | 7 |  |  |  | |  |
|  | 81.5 | 2.3 | L | Area hOc5 [V5/MT]  Inferior Occipital Gyrus | 101.4 | 8.73 | -40 | -72 | | -4 |
|  | 70.1 | 1.9 | L | Area hOc3d [V3d] | 7.1 |  |  |  | |  |
|  | 67.5 | 1.9 | L | Area hOc1 [V1] | 3.3 |  |  |  | |  |
|  | 20.6 | 0.6 | L | Area FG1 | 8.1 |  |  |  | |  |
|  | 18.6 | 0.5 | L | Area hOc2 [V2] | 2 |  |  |  | |  |
|  | 11.4 | 0.3 | L | Area hOc4d [V3A] | 2 |  |  |  | |  |
|  | 4.9 | 0.1 | L | Area FG4 | 0.8 |  |  |  | |  |
|  | 3 | 0.1 | L | Area PGp (IPL) | 0.4 |  |  |  | |  |
| 3352 | 448.4 | 448.4 | R | Area 45  R IFG (p. Opercularis) | 43.4 | 7.29 | 56 | 18 | | 32 |
|  |  |  | R | Area 45  IFG (p. Triangularis) |  | 6.81 | 52 | 20 | | 24 |
|  | 256.5 | 256.5 | R | Area 44  R IFG (p. Opercularis) | 42.7 | 7.88 | 58 | 16 | | 6 |
|  | 13 | 13 | R | Area Fo2 | 1.2 |  |  |  | |  |
|  | 8 | 8 | R | Area Id1 | 4.9 |  |  |  | |  |
|  | 1.4 | 1.4 | R | Thal: Parietal | 0.4 |  |  |  | |  |
|  | 0.8 | 0.8 | R | Thal: Temporal | 0.1 |  |  |  | |  |
|  |  |  | R | Insula Lobe |  | 9.33 | 38 | 20 | | -2 |
|  |  |  | R | Insula Lobe |  | 6.30 | 28 | 20 | | -16 |
|  |  |  | R | Middle Frontal Gyrus |  | 7.25 | 46 | 48 | | 4 |
|  |  |  | R | Precentral Gyrus |  | 6 | 46 | 6 | | 36 |
| 2850 | 401.6 | 14.1 | R | Area hOc4la | 45.3 |  |  |  | |  |
|  | 252 | 8.8 | R | Area hOc4lp  Middle Temporal Gyrus | 45 | 8.16 | 44 | -72 | | 2 |
|  |  |  | R | Area hOc4lp  Middle Occipital Gyrus |  | 6.69 | 46 | -78 | | 6 |
|  | 202.1 | 7.1 | R | Lobule VI (Hem) | 11.2 |  |  |  | |  |
|  | 181.8 | 6.4 | R | Lobule VIIa crusI (Hem) | 5.6 |  |  |  | |  |
|  | 112 | 3.9 | R | Area FG3  Inferior Temporal Gyrus | 17.1 | 6.66 | 42 | -56 | | -14 |
|  | 81 | 2.8 | R | Area hOc4v [V4(v)] | 13 |  |  |  | |  |
|  | 79.5 | 2.8 | R | Area FG2 | 24.4 |  |  |  | |  |
|  | 56.8 | 2 | R | Area hOc5 [V5/MT]  Middle Temporal Gyrus | 97.4 | 8.91 | 46 | -66 | | 4 |
|  | 44.9 | 1.6 | R | Area hOc3v [V3v] | 5.3 |  |  |  | |  |
|  | 31.9 | 1.1 | R | Area FG1 | 12.8 |  |  |  | |  |
|  | 21.3 | 0.7 | R | Area hOc3d [V3d] Middle Occipital Gyrus | 3.9 | 5.94 | 30 | -94 | | 6 |
|  | 11.9 | 0.4 | R | Area hOc4d [V3A] | 2.8 |  |  |  | |  |
|  | 10.5 | 0.4 | R | Lobule VI (Verm) | 4.5 |  |  |  | |  |
|  | 5.9 | 0.2 | R | Area hOc1 [V1] | 0.3 |  |  |  | |  |
|  | 5.9 | 0.2 | R | Area FG4 | 1.2 |  |  |  | |  |
|  | 5.6 | 0.2 | R | Area PGp (IPL) | 0.6 |  |  |  | |  |
|  | 3.5 | 0.1 | R | Area PGa (IPL) | 0.5 |  |  |  | |  |
|  |  |  | R | Inferior Temporal Gyrus |  | 9.15 | 44 | -68 | | -4 |
| 1854 | 371 | 20 | L | Thal: Prefrontal | 58.8 | 6.48 | 4 | -6 | | 2 |
|  | 303.5 | 16.4 | R | Thal: Prefrontal | 54.2 | 5.74 | 14 | -2 | | 6 |
|  |  |  | R | Thal: Prefrontal  Thalamus |  | 5.54 | 10 | -8 | | 6 |
|  | 142.5 | 7.7 | R | Thal: Temporal | 26.1 | 5.92 | -12 | -8 | | 6 |
|  |  |  | R | Thal: Temporal  Thalamus |  | 5.50 | 8 | -14 | | 10 |
|  | 93.4 | 5 | L | Thal: Temporal | 17.6 | 5.54 | -2 | -8 | | 2 |
|  | 15.3 | 0.8 | L | Thal: Premotor | 12.9 |  |  |  | |  |
|  | 1.4 | 0.1 | R | Thal: Parietal | 0.4 |  |  |  | |  |
|  | 0.3 | 0 | L | Thal: Parietal | 0.1 |  |  |  | |  |
|  |  |  | L | Pallidum |  | 5.97 | -12 | 2 | | -2 |
| 1227 | 252 | 20.5 | R | Area hIP3 (IPS)  Inferior Parietal Lobule | 55.2 | 5.36 | 34 | -54 | | 48 |
|  | 154 | 12.6 | R | Area PF (IPL)  SupraMarginal Gyrus | 22.8 | 5.30 | 64 | -32 | | 24 |
|  | 153.1 | 12.5 | R | Area PFm (IPL)  SupraMarginal Gyrus | 21.7 | 6.12 | 54 | -44 | | 42 |
|  | 107.6 | 8.8 | R | Area PFcm (IPL) Superior Temporal Gyrus | 33 | 5.30 | 60 | -36 | | 20 |
|  | 89.1 | 7.3 | R | Area 7PC (SPL)  Superior Parietal Lobule | 19.6 | 5.95 | 34 | -54 | | 60 |
|  |  |  | R | Area 7PC (SPL)  Inferior Parietal Lobule |  | 4.89 | 32 | -46 | | 50 |
|  | 71 | 5.8 | R | Area PFt (IPL) | 17 |  |  |  | |  |
|  | 57.6 | 4.7 | R | Area PFop (IPL)  SupraMarginal Gyrus | 25.2 | 4.55 | 58 | -20 | | 26 |
|  | 48.1 | 3.9 | R | Area 2 | 7.4 |  |  |  | |  |
|  | 41.9 | 3.4 | R | Area hIP2 (IPS) | 19.9 |  |  |  | |  |
|  | 26.1 | 2.1 | R | Area hIP1 (IPS) | 9 |  |  |  | |  |
|  | 22.6 | 1.8 | R | Area 7A (SPL) | 2.9 |  |  |  | |  |
|  | 4.5 | 0.4 | R | Area PGa (IPL) | 0.6 |  |  |  | |  |
|  | 0.9 | 0.1 | R | Area 1 | 0.1 |  |  |  | |  |
|  | 0.6 | 0.1 | R | Area OP1 [SII] | 0.2 |  |  |  | |  |
|  | 0.6 | 0.1 | R | Area 3b | 0.1 |  |  |  | |  |
|  | 0.3 | 0 | R | Area 3a | 0.1 |  |  |  | |  |
| 947 |  |  | L | Superior Medial Gyrus |  | 6.98 | 2 | 32 | | 50 |
|  |  |  | R | ACC |  | 6.70 | 8 | 34 | | 20 |
|  |  |  | R | Posterior-Medial Frontal |  | 6.44 | 4 | 22 | | 58 |
|  |  |  | R | Superior Medial Gyrus |  | 6.44 | 4 | 30 | | 42 |
|  |  |  | L | ACC |  | 4.95 | 0 | 30 | | 30 |
| 933 |  |  | L | PCC |  | 6.15 | -8 | -42 | | 24 |
| 705 |  |  | L | Insula |  | 8.98 | -30 | 18 | | -10 |
|  |  |  | L | IFG (p. Triangularis) |  | 5.89 | -42 | 20 | | 8 |
| 278 | 118 | 42.4 | L | Area hIP3 (IPS)  Inferior Parietal Lobule | 25.8 | 5.72 | -34 | -46 | | 48 |
|  | 26.3 | 9.4 | L | Area hIP1 (IPS) | 7.2 |  |  |  | |  |
|  | 18.5 | 6.7 | L | Area 7PC (SPL) | 10.8 |  |  |  | |  |
|  | 1.8 | 0.6 | L | Area 7A (SPL)  Superior Parietal Lobule | 0.1 | 3.81 | -30 | -56 | | 56 |
|  | 1.1 | 0.4 | L | Area 5L (SPL) | 0.2 |  |  |  | |  |
|  | 0.5 | 0.2 | L | Area 2 | 0.1 |  |  |  | |  |
| 162 |  |  | L | Precuneus |  | 4.65 | -10 | -60 | | 32 |
| 160 |  |  | R | Middle Temporal Gyrus |  | 5.06 | 56 | -30 | | -8 |
