# Supplementary material for: Commanding or Being a Simple Intermediary: How Does It Affect Moral Behavior and Related Brain Mechanisms?
Source: eNeuro. 2022 Oct 14;9(5):ENEURO.0508-21.2022. doi: 10.1523/ENEURO.0508-21.2022 (PMC9581580; doi:10.1523/ENEURO.0508-21.2022)
Supplement: Figure 4-1 — BOLD activity of comparisons between the agent and commander study conditions. Only clusters surviving a 5% FWE correction at the cluster size are reported (t = 3.5; p < 0.001; cluster size, 160). Brain regions are identified using the Anatomy Toolbox (Eickhoff et al. 2005). Download Figure 4-1, DOCX file. [file enu-eN-CFN-0508-21-s03.docx]

**Extended Data Figure 4-1. BOLD activity of comparisons between the agent and commander study conditions.** Only clusters surviving a 5% FWE correction at the cluster size are reported (t=3.5, p < .001, cluster size 160). Brain regions are identified using the Anatomy Toolbox (Eickhoff et al. 2005).

| **Cluster size** | **Voxels in cyto** | **% Cluster** | **Hem** | **Cyto or Anatomical description** | **% Area** | **Peak**  **t-value** | **MNI coordinates** | | |
| --- | --- | --- | --- | --- | --- | --- | --- | --- | --- |
|  |  |  |  |  |  |  | **x** | **y** | **z** |
| **Two sample t-tests between**  **Agent Free Shocks - Noshock and Intermediary with Human Agent Shocks - Noshock**  (5% FWE correction . t=3.5 . p<.001 . k=236) | | | | | | | | | |
| 373 | 97 | 26 | L | Area PFt (IPL)  Postcentral Gyrus | 16.6 | 4.43 | -52 | -22 | 28 |
|  |  |  | L | Area PFt (IPL)  SupraMarginal Gyrus |  | 3.59 | -62 | -26 | 32 |
|  | 95.6 | 25.6 | L | Area PFop (IPL)  Postcentral Gyrus | 43.1 | 4.48 | -58 | -20 | 26 |
|  | 66.3 | 17.8 | L | Area PF (IPL)  SupraMarginal Gyrus | 12.7 | 4.74 | -64 | -32 | 28 |
|  | 27 | 7.2 | L | Area PFcm (IPL) | 8.3 |  |  |  |  |
|  | 15.4 | 4.1 | L | Area 3b  Postcentral Gyrus | 2.7 | 4.37 | -48 | -22 | 32 |
|  | 12 | 3.2 | L | Area 2 | 2.3 |  |  |  |  |
|  | 4.9 | 1.3 | L | Area OP1 [SII] | 1.3 |  |  |  |  |
|  | 4.1 | 1.1 | L | Area 3a | 1.4 |  |  |  |  |
|  | 1.8 | 0.5 | L | Area OP4 [PV] | 0.5 |  |  |  |  |
|  | 1.1 | 0.3 | L | Area TE 3 | 0.1 |  |  |  |  |
|  | 0.5 | 0.1 | L | Area 1 | 0.1 |  |  |  |  |
|  |  |  | L | Superior Temporal Gyrus |  | 3.51 | -62 | -40 | 14 |
| 237 | 50.3 | 21.2 | R | Area PFcm (IPL)  Superior Temporal Gyrus | 15.4 | 3.63 | 60 | -32 | 18 |
|  | 39 | 16.5 | R | Area PF (IPL)  Superior Temporal Gyrus | 5.8 | 4.48 | 60 | -40 | 18 |
|  | 37.5 | 15.8 | R | Area PFm (IPL) | 5.3 |  |  |  |  |
|  | 7.9 | 3.3 | R | Area PGa (IPL) | 1.1 |  |  |  |  |
|  |  |  | R | Middle Temporal Gyrus |  | 3.66 | 60 | -46 | 10 |
| 236 | 7.5 | 3.2 | R | Area 44 | 1.2 |  |  |  |  |
|  | 0.4 | 0.2 | R | Area 45 | 0 |  |  |  |  |
|  |  |  | R | IFG (p. Opercularis) |  | 4.81 | 46 | 8 | 28 |
|  |  |  | R | Precentral Gyrus |  | 4.03 | 48 | 6 | 42 |
| **Two sample t-tests between**  **Agent Free Shocks - Noshock and Commander of Human Agent Shocks - Noshock**  (5% FWE correction . t=3.5 . p<.001 . k=163) | | | | | | | | | |
| 258 | 33.3 | 12.9 | R | Area FG4  Fusiform Gyrus | 6.8 | 4.48 | 30 | -52 | -10 |
|  | 5 | 1.9 | R | Lobule V (Hem) | 0.6 |  |  |  |  |
|  | 3.4 | 1.3 | R | Subiculum | 0.9 |  |  |  |  |
|  | 2.6 | 1 | R | Area FG1 | 1.1 |  |  |  |  |
|  | 0.9 | 0.3 | R | Area hOc3v [V3v] | 0.1 |  |  |  |  |
|  | 0.4 | 0.1 | R | Area hOc1 [V1] | 0 |  |  |  |  |
|  | 0.3 | 0.1 | R | Area hOc2 [V2] | 0 |  |  |  |  |
|  |  |  | R | Fusiform Gyrus |  | 4.86 | 26 | -42 | -12 |
|  |  |  | R | ParaHippocampal Gyrus |  | 3.90 | 18 | -42 | -6 |
|  |  |  | R | Cerebelum (IV-V) |  | 3.80 | 22 | -36 | -20 |
| 232 | 75.9 | 32.7 | L | Area FG4  Fusiform Gyrus | 12.8 | 5.59 | -26 | -52 | -10 |
|  | 33 | 14.2 | L | Area hOc1 [V1]  Lingual Gyrus | 1.6 | 4.04 | -14 | -58 | -4 |
|  | 10.3 | 4.4 | L | Area hOc2 [V2] | 1.1 |  |  |  |  |
|  | 5 | 2.2 | L | Area hOc3v [V3v] | 0.5 |  |  |  |  |
|  | 1.1 | 0.5 | L | Area hOc4v [V4(v)] | 0.2 |  |  |  |  |
| 163 | 23 | 14.1 | R | Area PF (IPL) | 3.4 |  |  |  |  |
|  | 22 | 13.5 | R | Area PFm (IPL)  Superior Temporal Gyrus | 3.1 | 4.20 | 62 | -42 | 20 |
|  | 7 | 4.3 | R | Area PGa (IPL) | 0.9 |  |  |  |  |
|  | 6.6 | 4.1 | R | Area PFcm (IPL) | 2 |  |  |  |  |
|  |  |  | R | Superior Temporal Gyrus |  | 4.02 | 60 | -46 | 14 |
|  |  |  | R | Middle Temporal Gyrus |  | 3.98 | 60 | -44 | 10 |
| **Two sample t-tests between**  **Agent Coerced Shocks - Noshock and Commander of Human Agent Shocks - Noshock**  (5% FWE correction . t=3.5 . p<.001 . k=315) | | | | | | | | | |
| 315 | 37 | 11.7 | R | Area 4a  Precentral Gyrus | 3.4 | 4.12 | 22 | -30 | 66 |
|  | 7.2 | 2.3 | L | Area 4a  Paracentral Lobule | 0.8 | 3.77 | -4 | -24 | 70 |
|  | 4.7 | 1.5 | R | Area 4p | 1.5 |  |  |  |  |
|  |  |  | R | Posterior-Medial Frontal |  | 5.22 | 12 | -20 | 66 |
|  |  |  | R | Postcentral Gyrus |  | 4.69 | 12 | -30 | 62 |
|  |  |  | R | Superior Frontal Gyrus |  | 4.08 | 18 | -10 | 66 |
