# Supplementary figures and images for: Commanding or Being a Simple Intermediary: How Does It Affect Moral Behavior and Related Brain Mechanisms?
Source: eNeuro. 2022 Oct 14;9(5):ENEURO.0508-21.2022. doi: 10.1523/ENEURO.0508-21.2022 (PMC9581580; doi:10.1523/ENEURO.0508-21.2022)

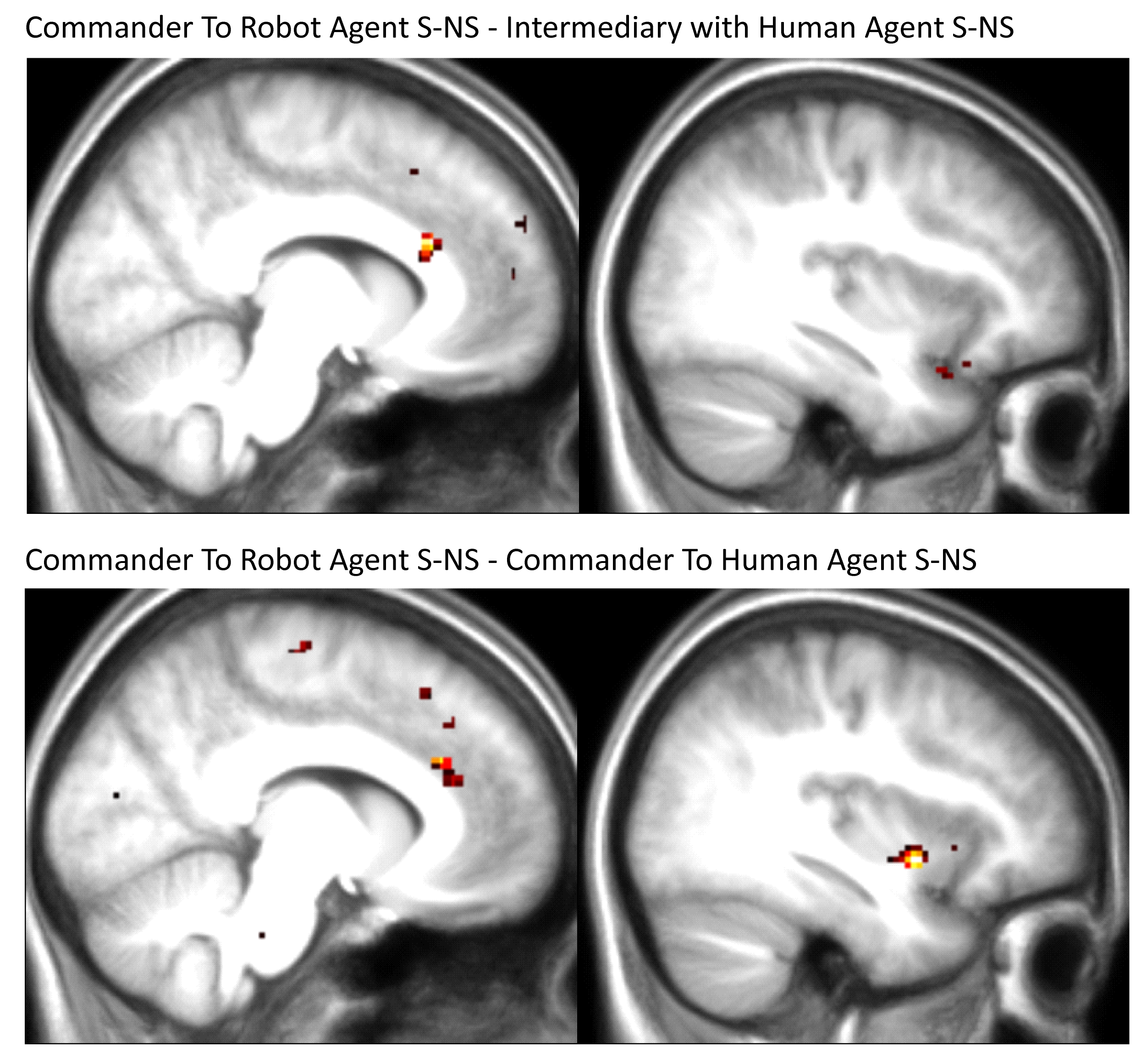

Supplement: Figure 4-2 — Results of two-sample t tests between the conditions CommanderOfRobotAgent(S-NS)–IntermediaryWithHumanAgent(S-NS) and CommanderOfRobotAgent(S-NS)–CommanderOfHumanAgent(S-NS) uncorrected (3.5 < t < 5; p < 0.005). Download Figure 4-2, TIF file. [file enu-eN-CFN-0508-21-s07.tif]

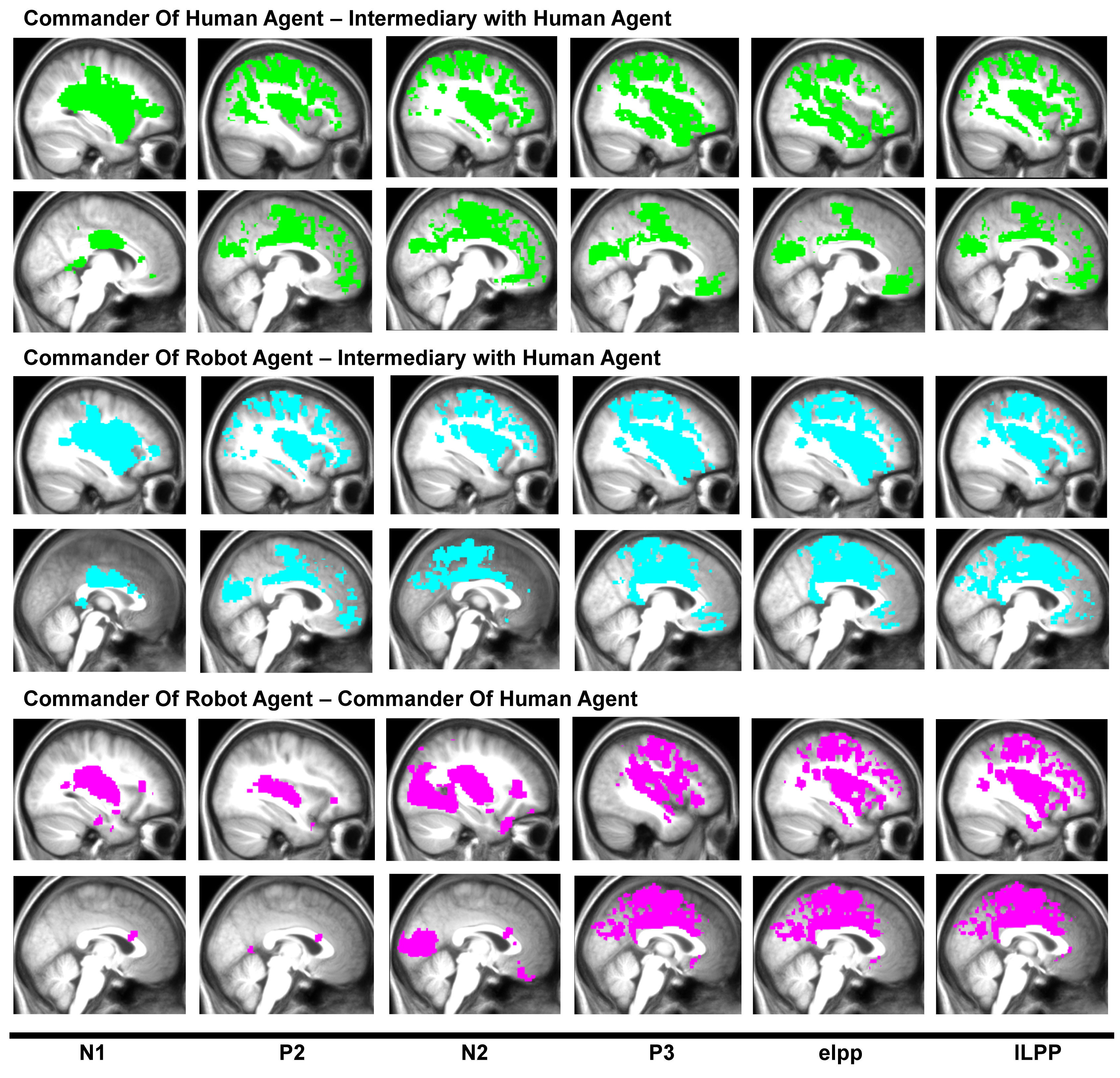

Supplement: Figure 8-2 — Maps derived reconstructing the EEG signal for the contrasts [CommanderOfHumanAgent(S-NS)–IntermediaryWithHumanAgent(S-NS)] in green, [CommanderOfRobotAgent(S-NS)–IntermediaryWithHumanAgent(S-NS)] in cyan, and [CommanderOfRobotAgent(S-NS)–CommanderOfHumanAgent(S-NS)] in violet. The voxels with the highest 5% of values encoding for regions that are the most positively involved in the selected ERP are displayed. Download Figure 8-2, TIF file. [file enu-eN-CFN-0508-21-s06.tif]

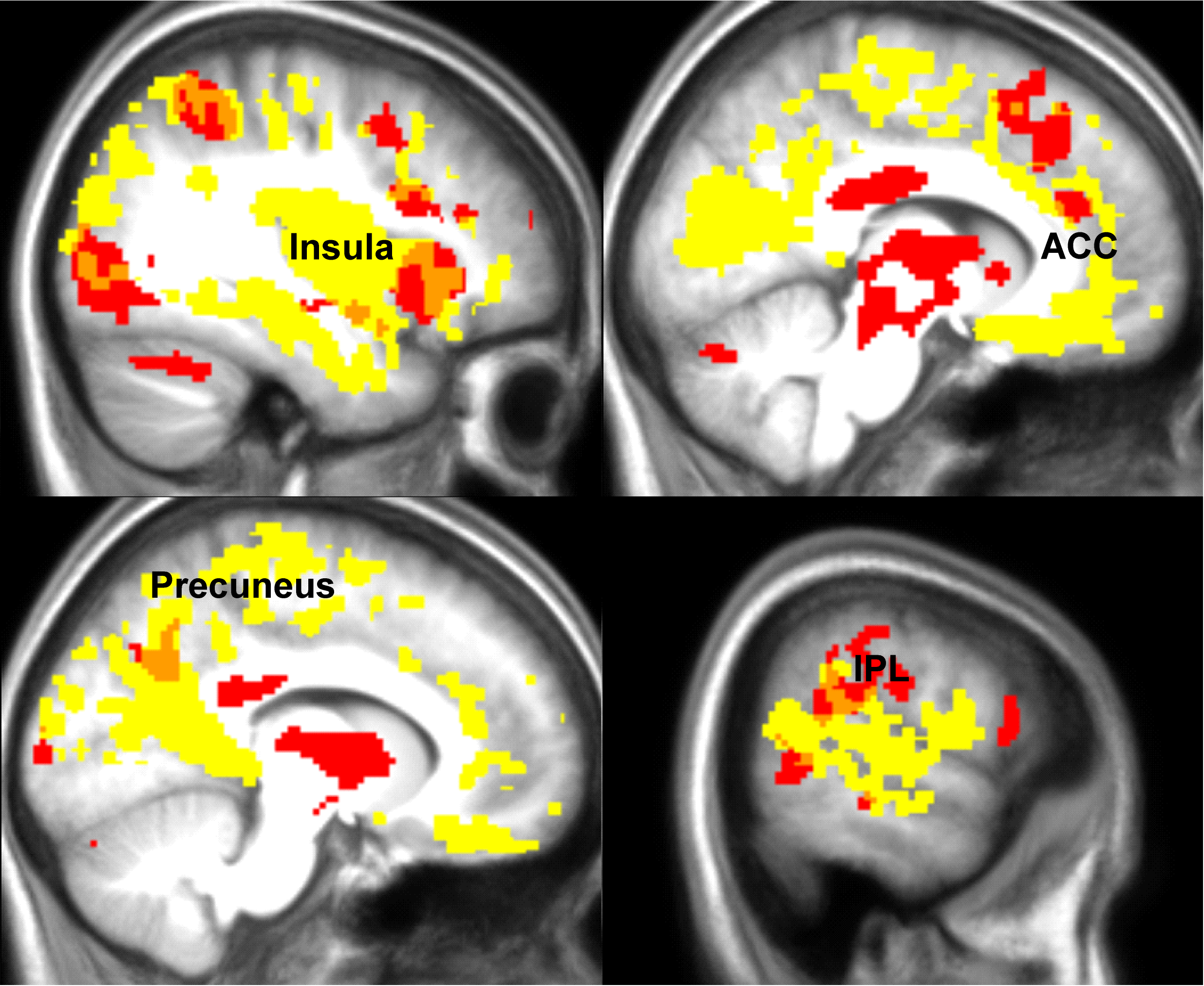

Supplement: Figure 8-3 — To compare Study 1 and Study 2, and to ensure that witnessing a shock versus no shock being delivered to the victim’s hand involve a similar brain activation pattern, we overlaid the MRI results obtained in Study 1 to the EEG results obtained in Study 2 for the Shock–No-Shock contrast. Overlap (in orange) is shown between the maps from the GLM analyses for Shock–No-Shock contrast (in red) and the 5% higher activation map derived reconstructing the EEG signal for the Shock–No-Shock contrasts using the mean activation from components P3, eLPP, and lLPP (in yellow). Download Figure 8-3, TIF file. [file enu-eN-CFN-0508-21-s05.tif]
