# Supplementary material for: Commanding or Being a Simple Intermediary: How Does It Affect Moral Behavior and Related Brain Mechanisms?
Source: eNeuro. 2022 Oct 14;9(5):ENEURO.0508-21.2022. doi: 10.1523/ENEURO.0508-21.2022 (PMC9581580; doi:10.1523/ENEURO.0508-21.2022)
Supplement: Figure 8-1 — Tables displaying the paired comparisons for Shock and No-Shock trials on Fz, Cz, and Pz. All tests were two tailed. Download Figure 8-1, DOCX file. [file enu-eN-CFN-0508-21-s02.docx]

**Extended Data Figure 8-1**. Tables displaying the paired comparisons for Shock and No shock trials on Fz, CZ and Pz. All tests were two-tailed.

Results on Fz

| **Comparisons** | **t** | **df** | ***p*** | **Cohen’s d** | **BF_10_** |
| --- | --- | --- | --- | --- | --- |
| P3 Pain – P3 No pain | -6.178 | 36 | <.001 | -1.016 | 40269.89 |
| eLPP Pain – eLPP No pain | -7.113 | 36 | <.001 | -1.169 | 581152.02 |
| lLPP – lLPP No pain | -5.747 | 36 | <.001 | -0.945 | 11713.97 |

Results on Cz

| **Comparisons** | **t** | **df** | ***p*** | **Cohen’s d** | **BF_10_** |
| --- | --- | --- | --- | --- | --- |
| P3 Pain – P3 No pain | -7.777 | 36 | <.001 | -1.279 | 3.760e+6 |
| eLPP Pain – eLPP No pain | -10.716 | 36 | <.001 | -1.762 | 8.642e+9 |
| lLPP – lLPP No pain | -7.919 | 36 | <.001 | -1.302 | 5.572e+6 |

Results on Pz

| **Comparisons** | **t** | **df** | ***p*** | **Cohen’s d** | **BF_10_** |
| --- | --- | --- | --- | --- | --- |
| **P3 Pain – P3 No pain** | -8.061 | 36 | <.001 | -1.325 | 8.252e+6 |
| **eLPP Pain – eLPP No pain** | -11.571 | 36 | <.001 | -1.902 | 6.831e+10 |
| **lLPP – lLPP No pain** | -8.786 | 36 | <.001 | -1.444 | 5.959e+7 |
